# Identification of a Lactate Accumulation Model to Explain the Heterogeneity in Prognosis, Immune Landscape, and Tumor Environment for HNSCC patients

**DOI:** 10.7150/jca.99560

**Published:** 2025-02-10

**Authors:** Yi Jin, Xiang Xiao, Jiayu Xiang, Tingjie Yu, Tingting Wang, Yonghong Zhou, Siwei Huang

**Affiliations:** 1 Department of Radiation Oncology, The Affiliated Cancer Hospital of Xiangya School of Medicine, Central South University/Hunan Cancer Hospital, Changsha, Hunan, 410013, China.; 2 Key Laboratory of Translational Radiation Oncology, Department of Radiation Oncology, Hunan Cancer Hospital and The Affiliated Cancer Hospital of Xiangya School of Medicine, Central South University, Changsha, 410013, China.; 3 The Affiliated Cancer Hospital of Xiangya School of Medicine, Central South University/Hunan Cancer Hospital, Changsha, Hunan, 410013, China;; 4 School of Medicine, Shanghai University, 99 Shangda Road, Shanghai 200444, China.; 5 School of Humanities and Management, Hunan University of Chinese Medicine, Changsha, Hunan 410208, China.

**Keywords:** head and neck squamous cell carcinoma, lactate accumulation, tumor-infiltrating immune-related lncRNA signature

## Abstract

Head and neck squamous cell carcinoma (HNSCC) is one of the most frequent cancers with a high mortality rate. Lactate accumulation, a hallmark of cancer, has received extensive attention, but its role in HNSCC remains underexplored. Therefore, we identified 33 prognostic genes related to lactate accumulation. By consensus clustering, we separated all HNSCC samples into cluster_A or cluster_B and explored the difference of clinicopathological characteristics and genomics landscape. Next, we performed LASSO analysis and RSF to calculate the lactate-related gene score (LRGS) and constructed a risk model with high accuracy for predicting survival, as estimated by ROC, nomogram, and calibration curve. Then, through OncoPredict algorithm and TCIA, we filter the suitable drugs, especially immunology with diverse LRGS. GSEA analysis showed that the DEGs of LRGS were enriched in activation of immune response and positive regulation of immune response. Moreover, we developed a tumor-infiltrating immune-related lncRNA signature (TILSig) through a combination of 115 immune cell lines from 16 GEO datasets and DealGPL570. Subsequently, we identified the 9 tumor-infiltrating immune-related lncRNAs and calculated the TIL_score. The correlations among these tumor-infiltrating immune-related lncRNAs, hub lactate-related genes and LRGS levels were visualized. According to validation using multiple datasets including TCGA, GSE65858, GSE41613, GSE27020, and the IMvigor 210 database, CARS2, NFU1, and SYNJ1 were identified as hub genes. In light of a comprehensive pan-cancer study, we analyzed these genes to detect the potential clinical value. In conclusion, the constructed LRGS provides important insights for subsequent mechanistic research and can guide clinicians in proposing therapeutic strategies for HNSCC patients.

## Background

Head and neck squamous cell carcinoma (HNSCC) is the eighth most frequent cancer in the world, with a high mortality rate, according to the Global Cancer Report of 2018 1. Currently, multidisciplinary treatments for HNSCC include surgery, chemotherapy, and radiotherapy 2. However, local and distant failures occur in up to 40% and 30% of patients, respectively 3. With an improved understanding of the molecular details, there has been a prominent surge in the field of anticancer drug therapy, resulting in new strategies to effectively treat many refractory cancers 4. Beginning with studies by Bonner and EXTREME, cetuximab was approved as the first molecular-targeted drug to prolong the median overall survival from 7.4 to 10.1 months in advanced HNSCC or recurrent/metastatic HNSCC 5,6. Subsequently, a deeper exploration of targeted immune checkpoint treatments was initiated, followed by the success of clinical trials 7. In 2019, immunologies, particularly pembrolizumab, were approved as the first-line treatment in the KEYNOTE-048 study 8. Nevertheless, due to the high heterogeneity, resistance, which results in worse outcomes after these novel treatments, has become a significant obstacle 9. The metabolic competition in the tumor environment may be regarded as a different clinical outcome of this heterogeneity, thereby developing a useful prognostic model to select proper treatment for HNSCC.

Lactate accumulation, a classic hallmark of cancer, is a waste byproduct of aerobic glycolysis during the Warburg effect. 10. Emerging results exhibit that lactate is essential for acidifying the tumor microenvironment (TME), accompanied by defective mitochondria and impaired adenosine triphosphate production to promote cell growth 11,12. This molecule can act as a fuel for mitochondrial procedures to reshape immune cell function and metabolism, inhibiting the activation and proliferation of immune cells to escape immune surveillance 13,14. Currently, lactate levels exceed the glucose concentration by 1.1- to 2.5-fold 15. The acute inflammatory response, such as arthritis and lactate accumulation, often amplifies inflammation or induces these underlying pathogenic conditions 16,17. One of the important processes of lactate accumulation is the lactate shuttles, which consist of intracellular and cell-cell shuttles 18, mediated by concentration gradients, pH gradients, and redox states. This process is transported across the plasma membrane by several monocarboxylate transporters (MCTs), mainly MCT1 and MCT4 19. An emerging theory was recently proposed that lactate homeostasis is the major source of cyclic carbohydrates for maintaining important systems 20. Moreover, a novel function of lactate, which plays a vital role in regulating gene expression through histone modifications, was discovered. This post-translational modification (PTM) of proteins is called lactylation.

Although lactate accumulation has received extensive attention, relevant studies are still limited. There have been no studies on lactate accumulation in patients with HNSCC. Accordingly, we used this study to explore innovations in customized precision diagnosis and treatment strategies for HNSCC.

## Materials and Methods

### Data acquisition and preprocessing

We downloaded HNSCC datasets from the Cancer Genome Atlas (TCGA) database (https://portal.gdc.cancer.gov/), including raw mRNA transcriptome profiles based on counts, copy number variation (CNV), and somatic mutation data. Next, normalized count data were derived using the DESeq2 package 21 and converted to TPM using the R function "counts_to_tpm." In this study, we also obtained clinical data, including TNM stage, gender, race, survival status, and survival time from TCGA as the training set. To validate the prognostic value of hub genes, the raw data of patients with HNSCC were retrieved from GEO under accession numbers GSE65858 and GSE41613 22,23 with data on overall survival (OS). Moreover, PFS data were obtained from GEO datasets under accession numbers GSE65858 and GSE27020 24. Based on the Molecular Signatures Database (MSigDB) (https://www.gsea-msigdb.org/gsea/msigdb), we identified nine classic lactate metabolism pathways, namely, total lactate transmembrane transport and lactate dehydrogenase activity, consisting of 324 genes. Then, we utilized the Cox analysis to select prognostic genes with *P* < 0.05, based on the TCGA-HNSC dataset for subsequent analysis.

### Consensus clustering of HNSCC subtypes

In this study, we excluded samples diagnosed with HNSCC with an OS > 30 days, and the survival time was recorded. "The R package "ConsensusClusterPlus" 25 was used to identify diverse subtypes in HNSCC, and K-means clustering, a method of vector quantization from signal processing to partition observations into k clusters, was conducted." The consensus matrix, cumulative distribution function (CDF), and relative change in the area under the CDF curve were employed to select the best clusters. Kaplan-Meier (KM) survival plots were used to calculate the OS rates of distinct clusters. Moreover, the distribution of TNM stage, somatic mutations, CNVs, TMB, and expression of related genes was visualized among diverse clusters. Finally, principal component analysis (PCA) was applied to validate the clustering results.

### Construction of a prognostic lactylation-related gene signature

First, we applied the least absolute shrinkage and selection operator (LASSO) to construct a model with the "glmnet" package 26. Then, the KM curves were utilized to exhibit the difference in OS between diverse subtypes, and we also used the package "survROC" to display the ROC curves 27. The random survival forest (RSF) has demonstrated superior predictive performance in risk model construction 28. Accordingly, we applied this method to determine essential genes based on cluster clustering or survival status to establish a risk model. Meanwhile, in both ways of the KM plot, the ROC curves were applied to validate the robustness of previous models. We identified the optimal method and generated the prognostic lactate-related gene score (LRGS) based on the validation results. Using univariate and multivariate Cox analysis, we compared the clinical value of the risk score with clinical features, such as the TNM stage in the TCGA dataset. Furthermore, a nomogram combining the LRGS and clinical characteristics was developed and assessed using calibration and ROC curves.

### Evaluation of drug sensitivity in HNSCC

A novel method developed by Maeser *et al.* 29, called OncoPredict, bridged *in vitro* drug screening with *in vivo* drug and biomarker discovery. Based on the statistics of the recorded 198 drugs, we selected the sensitivity of the drugs (between high- and low-LRGS groups or diverse clusters) with a threshold of *P* < 0.05.

### Exploration of the immune characteristics in HNSCC

Using the edgeR R package, we obtained differentially expressed genes (DEGs) between the diverse clusters with *P* < 0.05 by comparing diverse risk scores or clusters. Furthermore, using the R packages the "GSVA" and "GSEABase," we analyzed the main biological functions and the relationship between HNSCC and the immune landscape following the "c5.v7.4. symbols.gmt" database. To quantify the proportion of immune cells in HNSCC, CIBERSORT converted the gene matrix to the relative proportions of 22 TIIC subtypes 30. The ESTIMATE algorithm was used to calculate the immune-related score 31. Moreover, we downloaded the immunophenoscores (IPS) of HNSCC from the TCIA database (https://tcia.at/) to predict the sensitivity of immunotherapy when comparing the IPS among diverse clusters. In this study, we used a new strategy to identify and construct a tumor-infiltrating immune-related lncRNA signature (TILSig) through integrative analysis of lncRNA, immune, and clinical profiles of 115 immune cell lines, 187 NSCLC cell lines, and 1533 patients with NSCLC 32. Finally, prognostic tumor-infiltrating immune-related lncRNAs were determined for subsequent analysis.

### Identification and validation of LRGS and hub genes through multi-omics and pan-cancer analysis

To validate the prognostic value of clinical survival and immunology decision-making in LRGS, we downloaded the raw data of GSE65858 and GSE41613 to validate OS and the data of GSE65858 and GSE41613 for PFS. Furthermore, we obtained data from IMvigor 210 (NCT01208652 and NCT02951767) using the R package IMvigor210CoreBiologies (http://research-pub.gene.com/IMvigor210CoreBiologies/). In pan-caner analysis, we evaluated the relationship between hub genes and immune characteristics in the TIMER database, a comprehensive resource for systematical analysis of immune infiltrates across diverse cancer types 33. Next, we downloaded mRNA expression profiles, CNV, somatic mutation, and correlative clinical data from 33 types of cancer samples, including 11,315 samples. Finally, we comprehensively depicted the landscape of survival, somatic mutations, and CNV of hub genes in 33 types of cancer.

### Cell lines and cell culture

Oral cancer cells (Cal27 and HN6) were obtained from ATCC and cultured in Dulbecco's modified Eagle's medium (Procell, Wuhan, China) supplemented with 10% fetal bovine serum (FBS) (Procell, Wuhan, China). The cells were incubated in a thermostatic incubator with 5% CO_2_.

### RNA interference, transfection, and qRT-PCR

siRNAs and negative controls (NC) were designed and acquired from GenePharma (Suzhou, Jiangsu, China). The following primers for CARS2 were used (Forward: GTGTACCTGAGGGTAACCGAA; Reverse: TTGCCGTTGAATAAGCGTTCC). Next, we constructed siRNAs targeting CARS2 (si-CARS2#1 and si-CARS2#2) for further experiments. The knockdown efficiency was determined. Total RNA was extracted from cultured cells using the TRIzol reagent (Takara). The relative mRNA expression level was detected using the 2^-△△CT^ method, with GAPDH as the internal loading control.

### CCK8, Colony formation, and Transwell assays

A total of 3000 cells were seeded in 96-well plates (LABSELECT, China) with 100 μL of medium. The cell proliferation rate was determined using CCK-8 assays (Biosharp, China) at 0, 24, 48, 72, and 96 h. Briefly, 10 μL of CCK-8 reagent was added to each well at the specified time. After 2 h of incubation at 37 ℃, the absorbance was measured at a wavelength of 450 nm. Next, 1000 cells were plated in a 6-well plate with 2 mL of complete medium, and the medium was replaced every three days. After 14 days, colonies were stained with 0.25% crystal violet. A total of 5 × 10^4^ cells suspended in serum-free medium were added into the upper chamber, and 600 μL of DMEM containing 20% FBS was added to the lower chamber. After approximately 24 h of incubation, the cells in the Transwell system were stained with 0.25% crystal violet. The cells that migrated across the membrane were imaged and counted.

### Statistical analysis

All statistical analyses in this study were performed using R software (version 4.1.3; https://www.r-project.org/), and a *P*-value < 0.05 was considered statistically significant for all analyses.

## Results

### Construction of lactate-related subtypes in HNSCC

**Figure 1:**
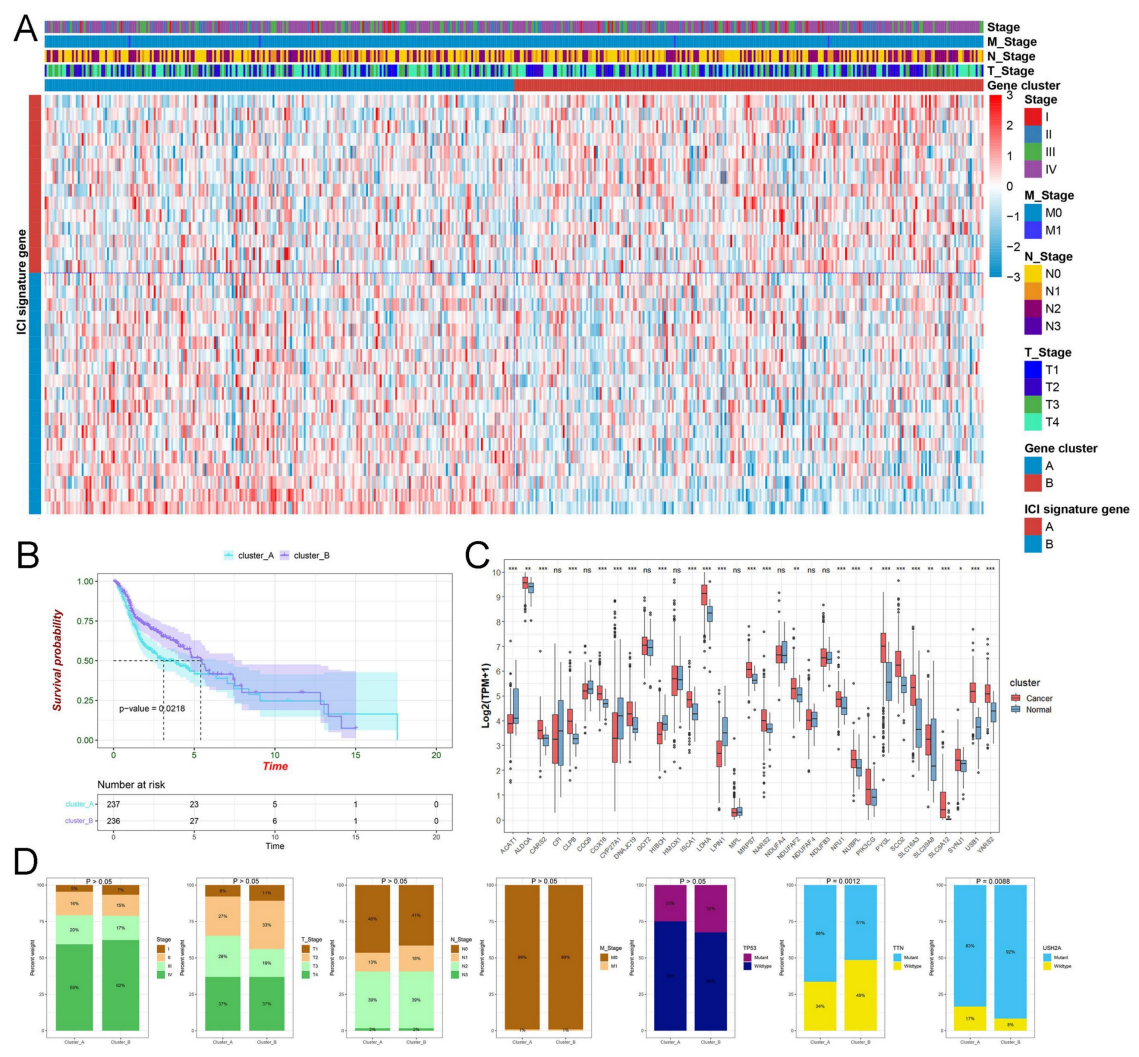
The clinical features of lactate-related subtypes in HNSCC. (A) The heatmap displays the gene expression of lactate-related genes and the distribution of clinicopathological characteristics. (B) Kaplan-Meier curves of OS for cluster_A and cluster_B. (C) The box plot displayed the expression of lactate-related genes in normal and HNSCC samples. (D) The barplot indicated the relationship between diverse clusters and T, N, M, clinical stage, and mutation status of TP53, TTN, respectively.

**Figure 2:**
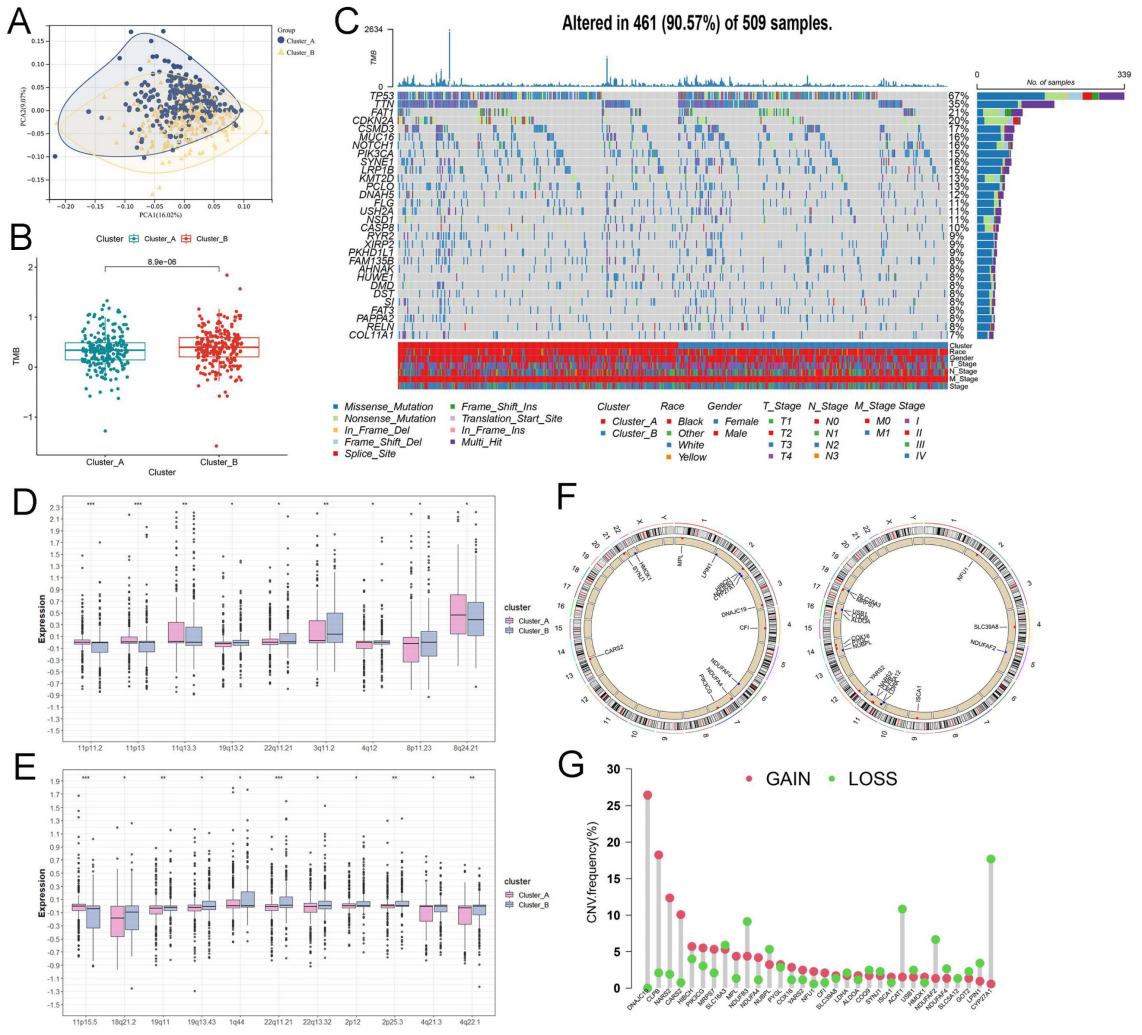
The genomics characteristics of lactate-related subtypes in HNSCC. (A) The PCA validated the clustering algorithm. (B) The difference of TMB in cluster_A and B. (C) The waterfall plot of top 30 mutated genes in HNSCC. (D) The amplification of CNA alterations between cluster_A and B showed significant differences (***P<0.001; **P<0.01; *P<0.05). (E) The difference in deletion of CNA between cluster_A and B (***P<0.001; **P<0.01; *P<0.05). (F) The location of important prognostic biomarkers. (G) The frequency of CNV, including gain and loss in cluster_A and B.

In this study, 500 HNSCC samples were downloaded from the TCGA database with the primary tumor, and 10 patients lacking follow-up information or survival of less than 30 days were excluded from survival analysis. In total, 324 lactate-related proteins from MSigDB were considered, and 34 genes were identified as potential prognostic biomarkers through Cox analysis in TCGA-HNSCC. Using consensus clustering with k = 2, we separated all HNSCC samples into two clusters, cluster_A and cluster_B **(Figure S1** and **Table S1)**. Next, we plotted the picture, including the TNM stage, diverse clusters, and lactate-related genes, into a heatmap **(Figure 1A)**. The KM plot illustrated that cluster_A had a much lower survival rate when compared to cluster_B with *P* = 0.0218 **(Figure 1B)**. The box plot displayed the prominent expressions of most prognostic lactylation-related genes in normal and HNSCC samples **(Figure 1C)**. Unfortunately, our findings in the barplot indicated that TNM stage DRG and most somatic mutations failed to exhibit significant differences in comparison of cluster_A and B except for TTN with *P =* 0.0012 and USH2A mutations with *P =* 0.0088** (Figure 1D)**. Additionally, we applied the PCA, a standard technique for statistical data analysis, to validate the clustering algorithm **(Figure 2A)**.

Interestingly, we observed a remarkable difference of TMB in cluster_A and B **(Figure 2B)**, implying the conspicuous genomic heterogeneity of somatic mutations and CNVs may play a significant role in HNSCC. First, the waterfall plot identified the top 30 most frequently mutated genes, such as TP53 with 67% mutation **(Figure 2C)**, and presented lactate-related somatic mutations in clusters_A and B, respectively **(Figure S2)**. Then, the boxplot presented the distribution of CNVs in cluster_A or B. As revealed in **Figure 2D**, many amplification regions of CNAs were enriched in the cluster_A, including 11p11.2, 11p13, 11q13.3, and 8q24.21, except for amplification-19q13.2, 22q11.21, 3q11.2, 4q12, and 8p11.23 **(Figure 2D)**. As for the distribution of deletion regions of CNAs, we observed that only deletion-11p15.5 was enriched in cluster_A **(Figure 2E)**. Based on the consensus clustering of cluster_A and B, we further analyzed the location of lactate biomarkers of two groups **(Figure 2F)** and visualized the frequency of CNVs **(Figure 2G)**.

### Establishment of lactate-related consensus signature

**Figure 3:**
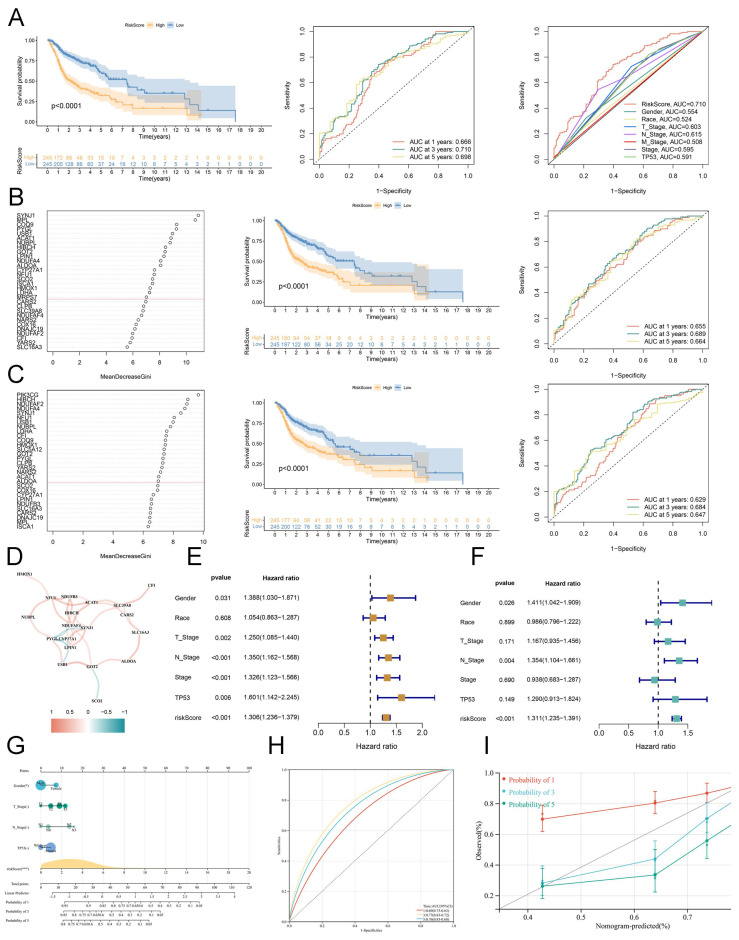
Establishment of lactate-related consensus signature. (A) The Kaplan-Meier curves of OS for high or low LRGS based on the LASSO analysis (left). The ROC analysis of 1 year, 3 years, and 5 years (median). The ROC analysis of LRGS and clinical features (right). (B) The variable importance plot for RFS model based on cluster_A and cluster_B (left). The Kaplan-Meier curves of OS for high or low LRGS based on the RSF analysis (median). The ROC analysis of 1 year, 3 years, and 5 years (right). (C) The variable importance plot for RFS model based on survival status (left). The Kaplan-Meier curves of OS for high or low LRGS based on the RSF analysis (median). The ROC analysis of 1 year, 3 years, and 5 years (right). (D) The possitive or negative interaction of lactate-related genes. (E) The univariate Cox analysis for clinical features and risk score. (F) The multivariate Cox analysis for clinical features and risk score. (G) The nomogram of risk model containing LRGS score and other clinicopathological features. (H) The ROC analysis of risk model from nomogram in 1 year, 3 years, and 5 years. (I) The calibration curve for validating the predictive efficacy of nomogram.

We performed LASSO regression to identify hub lactate-related biomarkers correlated with OS, and only 10 LRGs were used to construct a risk model **(Figure S3A)**. Based on this coefficient, we calculated the LRGS **(Table S2)** and obtained the LRGS of every patient with HNSCC. Selecting the median value as a cut-off, the KM analysis indicated that patients with a high LRGS score had shorter OS than others. Moreover, the area under the curve (AUC) value of 1-, 3-, and 5 years for ROC analysis was 0.666, 0.710, and 0.698, which suggests superior prognostic efficacy than other clinical features, including TNM stage, race, gender, and TP53 **(Figure 3A)**. The RSF is a marvelous method designed to handle survival data. First, we utilized this method to filter the biomarkers based on diverse clusters (cluster_A and B). As depicted in the KM survival curve, high-risk patients exhibited a worse prognosis compared to low-risk patients. Moreover, ROC analysis indicated that LRGS was an excellent biomarker compared to other clinical characteristics **(Figures 3B** and** S3B)**.

Furthermore, we found similar results when applying RSF to construct a risk model based on survival status **(Figures 3C** and** S3C)**. Combining these results above, these enrolled lactate genes intimately interacted with each other and mediated the tumor environment of HNSCC to influence the prognosis **(Figure 3D)**. Moreover, univariate and multivariate regression analyses demonstrated that LRGS was an independent risk factor for OS (hazard ratio = 1.311, 95% confidence interval (1.235-1.391); *P* < 0.001) **(Figures 3E-F)**. A nomogram that integrated the LRGS scores and other clinicopathological features was established **(Figure 3G)**. Then, we further estimated the survival rate of 1-, 3-, and 5-years based on the total points. Subsequently, the time-dependent ROC curve **(Figure 3H)** and calibration curve **(Figure 3I)** both indicated that this nomogram had high accuracy for predicting survival.

### Evaluation of the differences of drug and PD1/PD-L1 sensitivity in HNSCC

**Figure 4:**
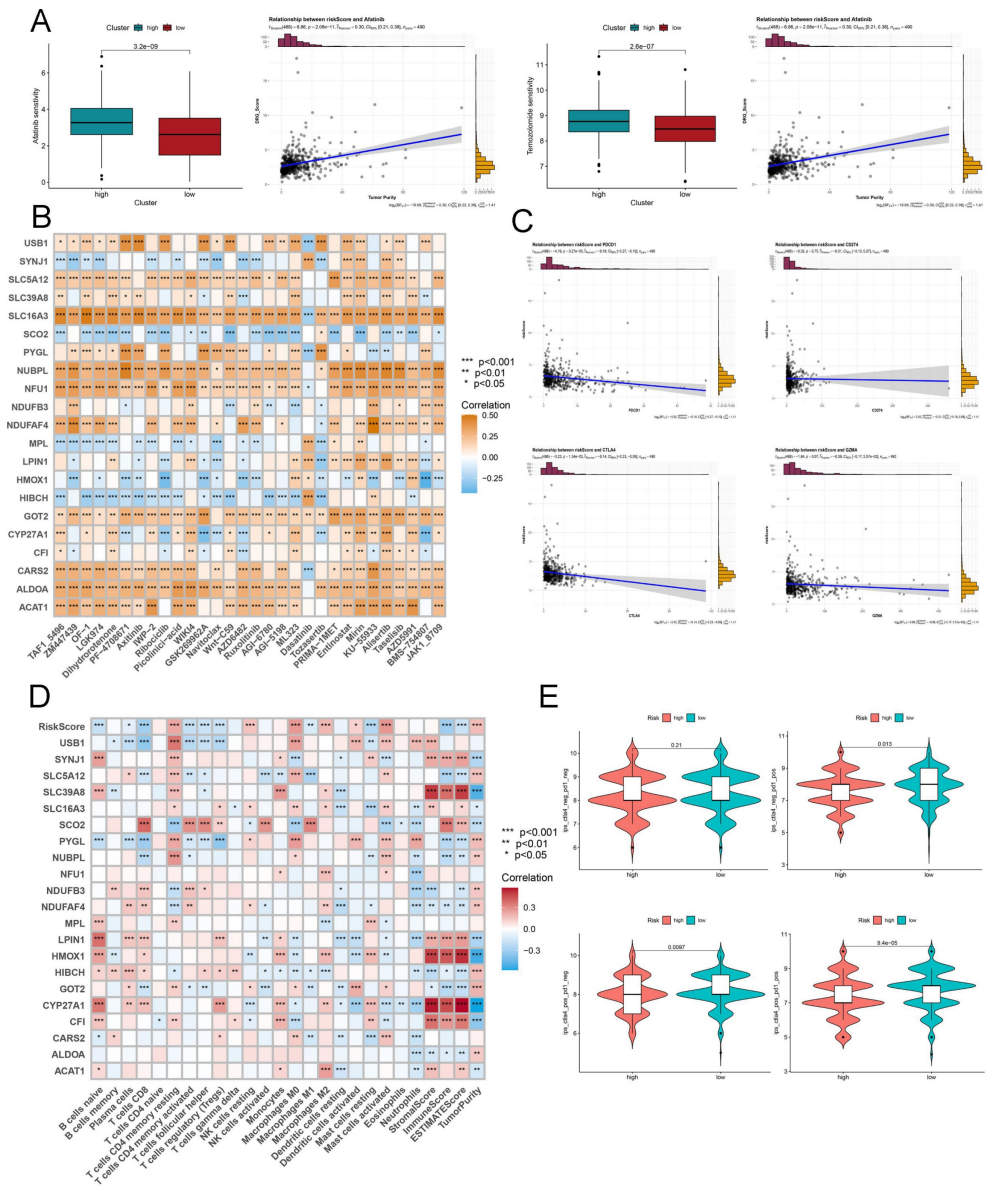
Evaluation the drug and PD1/PD-L1 sensitivity based on LRGS. (A) The drug sensitivity in low or high LRGS using the OncoPredict algorithm. (B) The correlation of hub lactate-related genes and drugs from OncoPredict. (C) The relationship between LRGS and PDCD1, CD274, CTLA4 and GZMA. (D) The correlation of hub lactate-related genes and immune cell types and immune-related scores from CIBERSORT and ESTIMATE. (E) The response to PD1/PD-L1 in high or low LRGS based on TCIA.

To explore suitable drugs for patients in cluster_A and B or low and high risk, HNSCC tissues from cluster_A exhibited greater resistance to seven drugs, including 5-fluorouracil, cisplatin, and docetaxel, than those from cluster_A patients except temozolomide **(Figure S4A)**. In view of LRGS, we found that afatinib and temozolomide presented less resistance in the high-risk group compared to the low-risk group **(Figure 4A)**. Next, we visualized the correlation between the hub lactate genes and multiple drugs with *P* < 0.05 **(Figure 4B)**. IPS values were calculated based on immunogenicity from the TCIA database. The outcome failed to depict the unambiguous relationship between diverse clusters and anti-PD-1/PD-L1 **(Figure S4B)**. To further investigate the quantification of TME indicators for individual patients with HNSCC by the methods of ESTIMATE and CIBERSORT, while in comparison of cluster_A and B, we also failed to unravel the difference of immune landscape **(Figure S5)**. Interestingly, LRGS was regarded as a novel and superior indicator of heterogeneity in the immune landscape compared to the previous clustering. First, we observed a negative association with LRGS and PDCD1, CTLA4** (Figure 4C)**. We then continuously displayed the relationship between hub lactate genes, immune cell types, and immune-related scores **(Figure 4D)**. Furthermore, following the data from TCIA, we presented the potential of diverse risk groups to respond to anti-PD-1/PD-L1 **(Figure 4E)**.

### Exploration of the innate mechanism of TME in HNSCC

**Figure 5:**
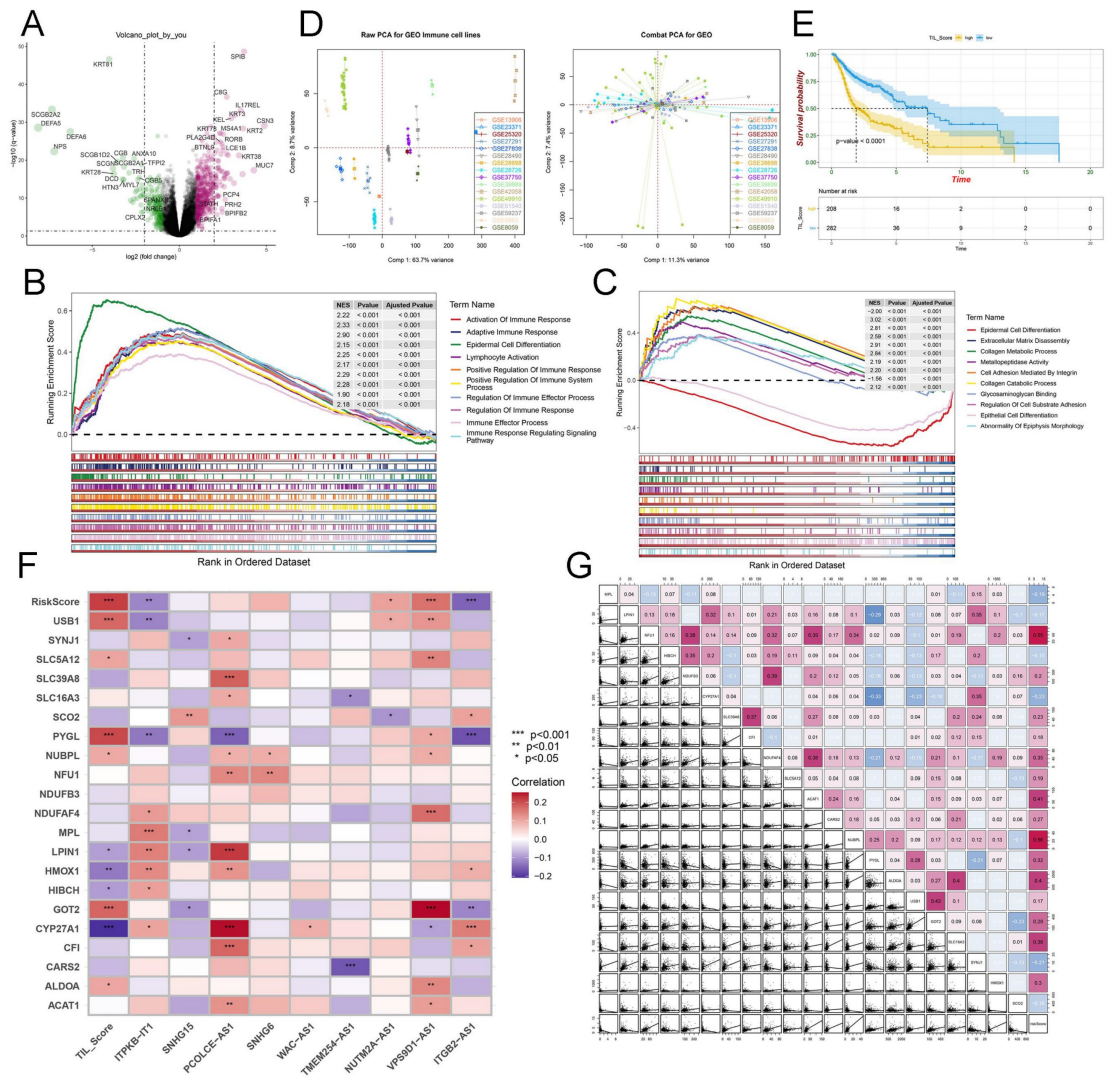
Innate mechanism exploration in HNSCC. (A) The volcano plot showing the DEGs based on LRGS. (B) The immune-related pathways from the GSEA enrichment analysis based on LRGS. (C) The main pathways from the GSEA enrichment analysis based on LRGS. (D) The raw PCA for GEO immune cell lines (left). The Combat PCA for GEO immune cell lines (right). (E) The Kaplan-Meier curves of OS for high or low TIL_score groups. (F) The association between tumor-infiltrating immune-related lncRNAs and lactate genes. (G) The innate correlation among lactate genes.

We identified up- and downregulated DEGs between the high- and low-risk groups (*P* < 0.05) **(Figure 5A)**. Furthermore, we utilized the "c5.v7.4.symbols.gmt" database to reveal that multiple pathways, such as immune response activation, adaptive immune response, and positive regulation of immune response and immune system process were activated by the high LRGS group **(Figure 5B)**. Besides, these DEGs participated in epidermal cell differentiation, extracellular matrix disassembly, epithelial cell differentiation, and glycosaminoglycan binding **(Figure 5C)**. Moreover, we obtained the DEGs based on clutser_A and B **(Figure S6A)** and presented the main pathways involved in the diverse clusters **(Figure S6B)**. In this study, we developed TILSig using a combination of 115 immune cell lines 32. We downloaded the raw data from 16 GEO datasets and utilized DealGPL570 to calculate the expression profiles of 114 cell lines and 19 immune cells. Then, through the R package "Combat," we integrated and calibrated all the data above **(Figure 5D)**. Subsequently, we identified nine important lncRNAs closely associated with tumor-infiltrating immune cells and calculated the TIL_score from these lncRNAs** (Table S3)**. Then, we performed a KM analysis to evaluate the influence of the TIL_score on survival, and the result demonstrated that the survival time of the high-TIL group was shorter than that of the low-TIL group **(Figure 5E)**. Furthermore, we plotted the association between lactate genes and tumor-infiltrating immune-related lncRNAs **(Figure 5F)** and the correlations between these hub lactate-related genes and LRGS levels in human HNSCC cancer tissues **(Figure 5G)**.

### Identification of the hub genes through multi-omics and pan-cancer analysis

**Figure 6:**
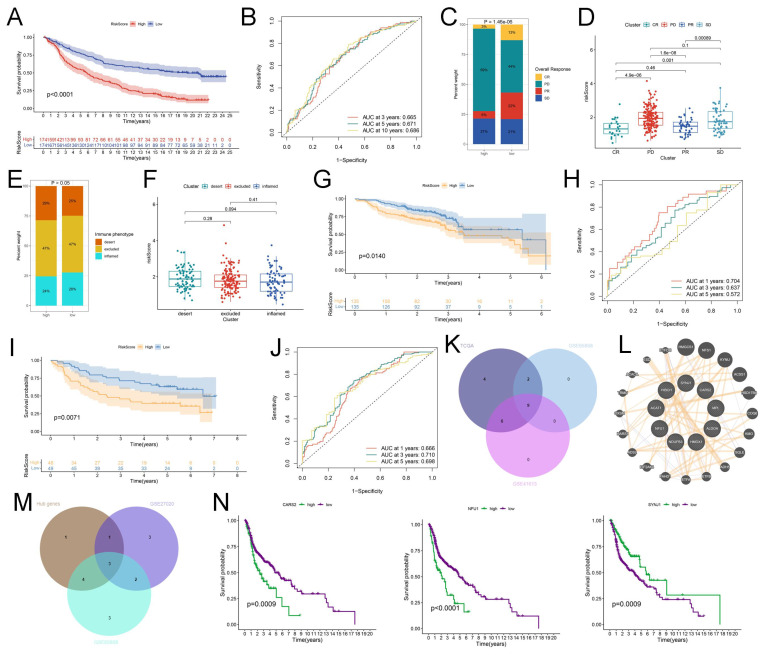
Identification and verification of the hub genes and LRGS. (A) The Kaplan-Meier curves of OS for high or low risk score groups in IMvigor 210. (B) The ROC analysis of 1 year, 3 years, and 5 years in IMvigor 210. (C) The barplot indicated the distribution of overall responses in high or low risk score groups. (D) The box plot displayed the expression of risk score in diverse overall responses. (E) The barplot showed the distribution of immune phenotypes in high or low risk score groups. (F) The box plot presented the expression of risk score in diverse immune phenotypes. (G) The Kaplan-Meier curves of OS for high or low risk score groups in GSE65858. (H) The ROC analysis of 1 year, 3 years, and 5 years in GSE65858. (I) The Kaplan-Meier curves of OS for high or low risk score groups in GSE41613. (J) The ROC analysis of 1 year, 3 years, and 5 years in GSE41613. (K) The Venn plot of prognostic LRGs of OS in TCGA, GSE65858 and GSE41613. (L) The Protein-protein interaction network of 9 hub genes from GeneMANIA. (M) The Venn plot of hub LRGs of OS in TCGA, prognostic LRGs of PFS in GSE65858 and LRGs of PFS in GSE27020. (N) The Kaplan-Meier curves of OS for CARS2, NFU1, and SYNJ1 in TCGA.

**Figure 7:**
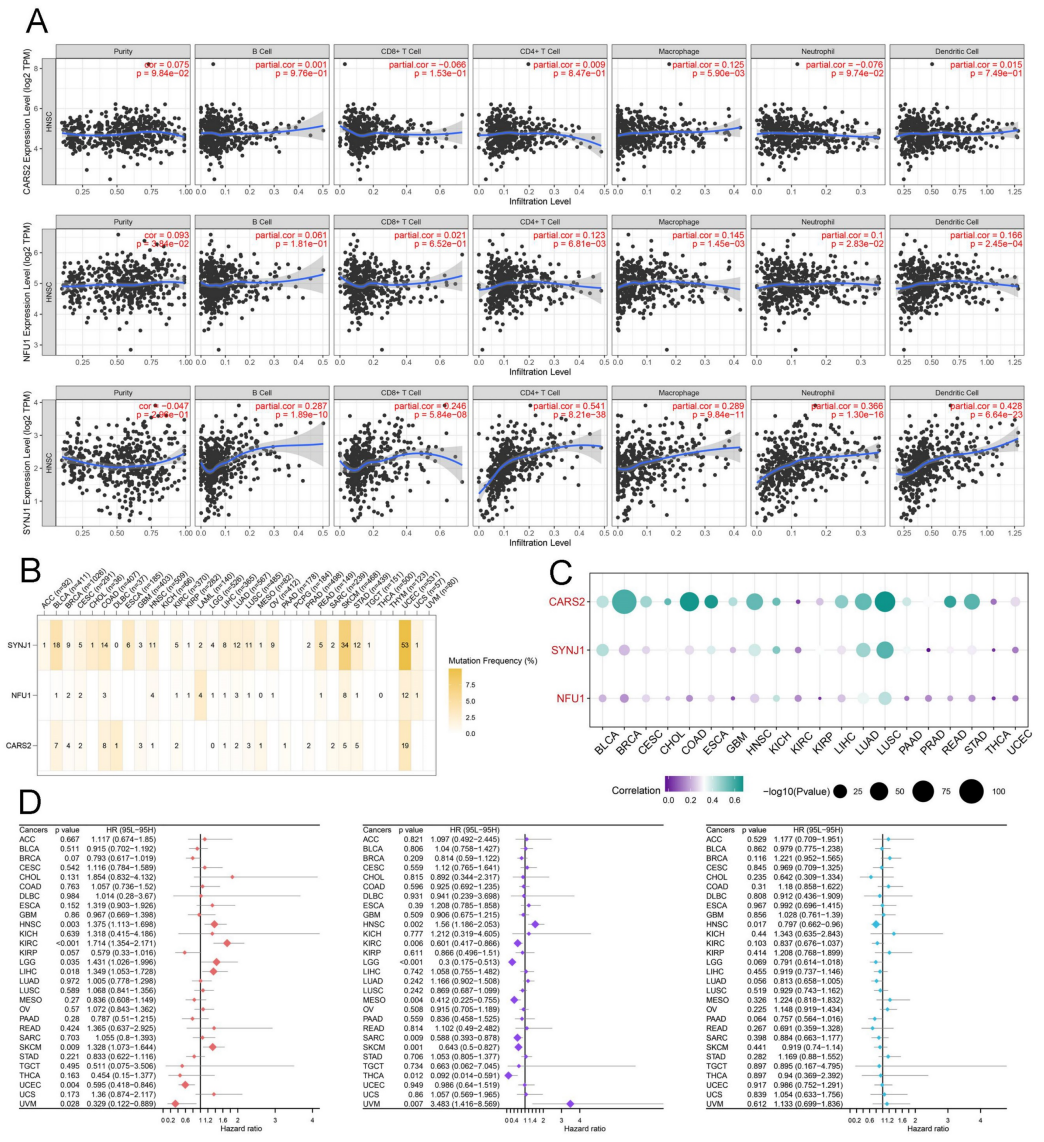
Assessment of the hub genes in pan-cancer analysis. (A) The association of expression of CARS2, NFU1, and SYNJ1 with immune cell infiltrations in HNSCC. (B) The correlation of variants frequency and the expression of CARS2, NFU1, and SYNJ in each cancer subtype. (C) The correlation of somatic copy number alterations and the expression of CARS2, NFU1, and SYNJ in each cancer subtype. (D) The univariate Cox regression of OS in CARS2 (left), NFU1 (median), and SYNJ1 (right) from the forest plot.

The IMvigor 210 database was used to analyze the immunotherapy response of these crucial lactate-related biomarkers. First, we performed LASSO regression to calculate the risk score, and KM analysis revealed a higher OS of the low-risk group in the IMvigor 210 database (*P* < 0.001) **(Figure 6A)**. Moreover, the AUC values at 1-, 3-, and 5-years were 0.665, 0.671, and 0.686, respectively **(Figure 6B)**. The percentages of the four immunotherapy response types (SD, PR, PD, and CR) were different between the two risk groups **(Figure 6C)**. The risk score was higher in the patients with PD/SD than in the patients with CR/PR **(Figure 6D)**. These data indicated that the low-risk group responded better to immunotherapy. However, there was no relationship between the risk score and immunophenotypes, including desert, excluded, and inflamed **(Figures 6E-F)**. Consequently, we believe that essential lactate-regulating genes do not participate in regulating immune phenotypes but rather have their roles. Further screening is needed for regulatory genes closely related to prognosis and immunity. To validate the prognostic value of hub genes and LRGS, we first downloaded the raw data of GSE65858 and GSE41613 for OS. The KM plot implied that LRGS was a potential indicator for predicting OS in GSE65858 **(Figure 6G)** and GSE41613 **(Figure 6I)**. Furthermore, the ROC curve was generated successfully to validate the ability of the logistic model to predict prognosis in GSE65858 **(Figure 6H)** and GSE41613 **(Figure 6J)**. Nine intersecting genes were revealed to act as hub genes in Venn plots, which are intimately related to OS **(Table S4** and** Figure 6K)**. The protein-protein interaction network of the nine hub genes was visualized using GeneMANIA **(Figure 6L)**. Next, we obtained PFS data from GEO datasets GSE65858 and GSE27020. The Venn plot filtered three hub genes: CARS2, NFU1, and SYNJ1 **(Table S4** and** Figure 6M)**. Finally, we drew the KM plots to present the prognostic ability of hub genes based on the TCGA **(Figure 6N)**, GSE65858 with OS **(Figure S7A)**, GSE41613 with OS **(Figure S7B)**, GSE27020 with PFS **(Figure S7C)**, GSE65858 with PFS **(Figure S7D)** and IMvigor 210 **(Figure S8A)**. However, we failed to observe a clear association between the three hub genes and immunotherapy response type **(Figure S8B)** or immunophenotype **(Figure S8C)**. Meanwhile, TIMER data illustrated the association between three hub gene expression and immune cell infiltration in HNSCC **(Figure 7A)**. Considering a comprehensive pan-cancer study, we analyzed the data for these three genes to detect the frequency of variants in each cancer subtype. As displayed in **Figure 7B**, mutation frequencies were quite high in multiple cancers, including skin cutaneous melanoma (SKCM) and uterine corpus endometrial carcinoma (UCEC) **(Figure 7B)**. Next, we depicted the relationship between somatic CNAs and the expression of the three hub genes in pan-cancer analysis **(Figure 7C)**. Finally, we presented forest plots to evaluate the prognostic roles of CARS2, NFU1, and SYNJ1 in multiple cancers **(Figure 7D)**.

### CARS2 regulated lactate and promote proliferation, migration, and invasion

**Figure 8:**
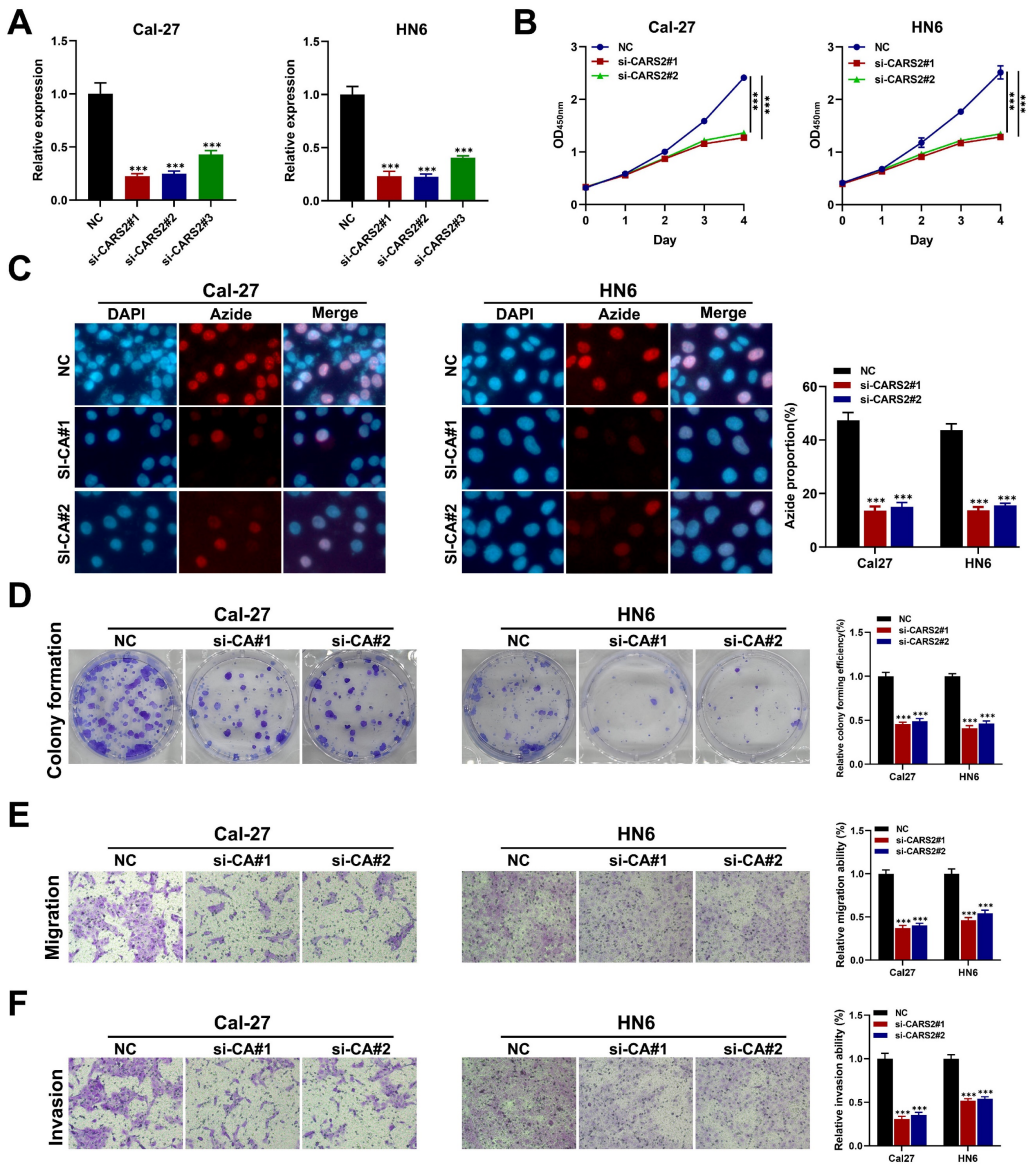
The validation of behavior of CARS2 on HNSCC (A) qRT-PCR in Cal27 and HN6. The CCK-8 (B), EdU assay (C), colony formation assay (D), and transwell assay, including migration ability (E) and invasion ability (F). ***P<0.001.

First, we downloaded data from GSE24922, including oral squamous cell carcinoma cell lines before and after lactate treatment, which was also validated in HNSCC tissues through ChIP-qPCR and RT-qPCR. Interestingly, we found that CARS2 played a significant role in lactate regulation in* in vitro* and *in vivo* experiments (**Table S5**). Moreover, we analyzed the relationship between hub genes and important clinical factors in HNSCC. CARS2 is an independent prognostic biomarker that predicts OS and PFS, and SYNJ1 and NFU1 may be related to different N stages that influence clinical outcomes (**Figure S9**). Further analysis revealed that CARS2 knockdown decreased its expression in Cal27 and HN6 cells **(Figure 8A)**. The CCK-8, EdU, colony formation, and transwell experiments presented that Cal27 and HN6 cell proliferation, migration, and invasion were inhibited by CARS2 knockdown **(Figures 8B-E)**. These results demonstrate that CARS2 promotes proliferation, invasion, and proliferation.

## Discussion

A classical metabolic phenomenon known as aerobic glycolysis is intimately related to the rapid proliferation of cancer cells, characterized by a high lactate production rate from glucose despite the availability of oxygen for mitochondrial respiration 34. Lactate accumulation is the first step in initiating aerobic glycolysis through four ways, including lactate exportion, shuttles, lactate homeostasis, and lactylation. In this study, we collected all genes related to lactate accumulation and calculated a lactate-related risk score, called LRGS, to unravel the association with clinical outcomes, such as OS and PFS, in patients with HNSCC. However, as demonstrated in our results, a single LRGS cannot perfectly predict the prognosis of HNSCC, and it does not have sufficient advantages over other models. This is because many specific subtypes of HNSCC are different from each other. Accordingly, our findings verify the role of LRGS in HNSCC, and further refinement is required. In this study, we also observed that LRGS can affect the efficiency of afatinib and temozolomide. Similar results have been reported for other cancers. Our findings suggest sensitization to afatinib therapy by metformin in TKI-resistant lung cancer cells and a reduction in cellular glycolytic phenotype 35. Furthermore, the glycolysis rate was accelerated by circKIF4A overexpression, which promoted glioma growth and temozolomide resistance 36.

It is a long-standing paradigm in which tumor cells employ aerobic glycolysis, which constitutes a target for anti-proliferative chemotherapies. Furthermore, metabolism is the basis of imaging by positron emission tomography. However, the role of these approaches in clinical practice is negligible to date 37. In 1985, the concept of intercellular lactate shuttling was proposed and systematically explained 38, which summarized the entire process of lactate transmembrane migration 39. Lactate is rapidly produced and accumulates in muscle cells at the start of exercise, some of which enters tissues and is then internalized and oxidized by adjacent cells.

In contrast, the remaining lactate enters the blood circulatory system, where it is a substrate for oxidative energy production and gluconeogenesis 40. With advances in research, lactate shuttling in different cell populations to regulate TME is a new phenomenon. Inspired by the discovery of Kac, in 2019, Zhang *et al.* first proposed histone K (L-la), a new type of PTM, to unravel a new for deeper dissection 41. To date, the roles of lactylation in regulating several processes in cancer development have been documented. Yu *et al.* found that increased levels of histone lactylation (H3K18la) were associated with poor prognosis in melanoma 42. Additionally, lactylation is intimately related to tumor-infiltrating immune cells. At the high levels of histone Kla, most TAM presented an M2 phenotype to contribute to the formation and progression of tumors 43.

In our study, LRGS was verified as a novel and superior indicator for explaining the heterogeneity in the immune landscape compared to other clustering methods based on lactate metabolism. We observed the remarkably negative association between LRGS and PDCD1 and CTLA4 and unraveled the relationship with significant immune cell types, such as CD8+ T cells, CD4+ T cells, macrophages M1 and M2. Previous literature has validated that lactic acid suppresses inflammatory macrophage (M1) function. Contrarily, it also enhances regulatory, or anti-inflammatory, M2 polarization 44,45. A mechanistic link between lactate, CD8+ T cell stemness, and improved cancer immunotherapy outcomes has been reported. In a prostate cancer model, lactate released by glycolytic CAFs acts on CD4+ T cells, shaping T-cell polarization and sustaining cancer malignancy 46. Therefore, lactate accumulation may lead to an abnormal distribution of immune cells, thereby altering the TME. For this reason, this study used TILSig to effectively analyze the immune microenvironment based on the TCGA public dataset. It is recognized that TILSig is a common algorithm that integrates a large amount of public database data from and verification of vitro experiments. Using this algorithm, we screened representative genes related to immunotherapy in HNSCC, evaluated the relationship between lactation genes and the genes above, and selected highly related HUB genes for follow-up research.

Through multi-omics and pan-cancer analyses, CARS2, NFU1, and SYNJ1 were identified as hub genes to construct LRGS and regulate the lactate-related TME in HNSCC. CARS2, also known as cysteinyl-tRNA synthetase 2, is a putative member of the class I family of aminoacyl-tRNA synthetases. Reportedly, CARS2 may produce cysteine persulfide (Cys-SSH) and maintain a stable level *in vitro*, thus stimulating mitochondrial ETC 47. Intriguingly, CARS2 mutation has exhibited neurological regression and mitochondrial dysfunction in epileptic encephalopathy 48. However, few studies have focused on the role of CARS2 in cancer development. In pan-cancer analysis, CARS2 acted as an oncogene in HNSCC, kidney renal clear cell carcinoma, liver hepatocellular carcinoma, and SKCM. In contrast, it was regarded as a suppressor gene in UCEC and uveal melanoma. NFU1, an iron-sulfur cluster scaffold, encodes a protein localized to the mitochondria and is critical in iron-sulfur cluster biogenesis. The abnormal function of NFU1 often causes a larger amount of mitochondrial dysfunction syndrome-1 49. According to a genome-wide CRISPR-Cas9 cell viability screen under physiological and acidic conditions, some researchers have systematically identified the important NFU1 gene associated with pH-related fitness defects in colorectal cancer cells 50. Moreover, the effect of different pH values, particularly at pH 5.2, may result in lactate accumulation 51. Consequently, we hypothesized that NFU1 participates in the mediation of pH concentration to regulate lactate accumulation, thereby interfering with HNSCC development. Synaptojanin 1 (SYNJ1) is a protein-coding gene related to developmental and epileptic encephalopathy and Parkinson's disease. In triple-negative breast cancer, mechanistic investigations demonstrated that LINC01234 might act as a competing endogenous RNA for miR-429 to upregulate SYNJ1 expression to induce cell proliferation and migration and impair cell apoptosis 52. However, no previous studies have focused on these hub genes in HNSCC.

This study has many shortcomings, including the lack of external verification of the model's reliability and stability. Second, basic *in vitro* and *in vivo* experiments are crucial for verifying many hypotheses. In this study, we comprehensively investigated LRG in the context of clinical characteristics and immune infiltration landscape in patients with HNSCC, guiding clinicians in proposing therapeutic strategies. Prospective research is essential to evaluate the clinical utility of the signature in patients with HNSCC.

## Supplementary Material

Supplementary figures and tables.

## Abbreviations

HNSCC: Head and neck squamous cell carcinoma
LRGS: Lactate-related gene score
TILSig: Tumor-infiltrating immune-related lncRNA signature
TME: Tumor microenvironment
MCT: Monocarboxylate transporters
PTM: Post-translational modification
TCGA: The Cancer Genome Atlas
CNV: Copy number variation
OS: Overall survival
MSigDB: Molecular Signatures Database
CDF: Cumulative distribution function
PCA: Principal component analysis
LASSO: Least absolute shrinkage and selection operator
RSF: Random survival forest
DEG: Differentially expressed gene
IPS: Immunophenoscores

## References

[1] Bray F, Ferlay J, Soerjomataram I (2018). Global cancer statistics 2018: GLOBOCAN estimates of incidence and mortality worldwide for 36 cancers in 185 countries. CA Cancer J Clin.

[2] Vokes EE (2010). Induction chemotherapy for head and neck cancer: recent data. Oncologist.

[3] Pulte D, Brenner H (2010). Changes in survival in head and neck cancers in the late 20th and early 21st century: a period analysis. Oncologist.

[4] Kitamura N, Sento S, Yoshizawa Y (2020). Current Trends and Future Prospects of Molecular Targeted Therapy in Head and Neck Squamous Cell Carcinoma. Int J Mol Sci.

[5] Bonner JA, Harari PM, Giralt J (2006). Radiotherapy plus cetuximab for squamous-cell carcinoma of the head and neck. N Engl J Med.

[6] Vermorken JB, Mesia R, Rivera F (2008). Platinum-based chemotherapy plus cetuximab in head and neck cancer. N Engl J Med.

[7] Mei Z, Huang J, Qiao B (2020). Immune checkpoint pathways in immunotherapy for head and neck squamous cell carcinoma. Int J Oral Sci.

[8] Burtness B, Harrington KJ, Greil R (2019). Pembrolizumab alone or with chemotherapy versus cetuximab with chemotherapy for recurrent or metastatic squamous cell carcinoma of the head and neck (KEYNOTE-048): a randomised, open-label, phase 3 study. Lancet.

[9] Kurten CHL, Kulkarni A, Cillo AR (2021). Investigating immune and non-immune cell interactions in head and neck tumors by single-cell RNA sequencing. Nat Commun.

[10] Koppenol WH, Bounds PL, Dang CV (2011). Otto Warburg's contributions to current concepts of cancer metabolism. Nat Rev Cancer.

[11] Rastogi S, Mishra SS, Arora MK (2023). Lactate acidosis and simultaneous recruitment of TGF-beta leads to alter plasticity of hypoxic cancer cells in tumor microenvironment. Pharmacol Ther.

[12] Chen S, Duan H, Sun G (2023). Reshaping immunometabolism in the tumour microenvironment to improve cancer immunotherapy. Biomed Pharmacother.

[13] Mu X, Xiang Z, Xu Y (2022). Glucose metabolism controls human gammadelta T-cell-mediated tumor immunosurveillance in diabetes. Cell Mol Immunol.

[14] Ganapathy-Kanniappan S (2017). Linking tumor glycolysis and immune evasion in cancer: Emerging concepts and therapeutic opportunities. Biochim Biophys Acta Rev Cancer.

[15] Hui S, Ghergurovich JM, Morscher RJ (2017). Glucose feeds the TCA cycle via circulating lactate. Nature.

[16] Haas R, Smith J, Rocher-Ros V (2015). Lactate Regulates Metabolic and Pro-inflammatory Circuits in Control of T Cell Migration and Effector Functions. PLoS Biol.

[17] Certo M, Tsai CH, Pucino V (2021). Lactate modulation of immune responses in inflammatory versus tumour microenvironments. Nat Rev Immunol.

[18] Brooks GA (2020). Lactate as a fulcrum of metabolism. Redox Biol.

[19] Sun S, Li H, Chen J (2017). Lactic Acid: No Longer an Inert and End-Product of Glycolysis. Physiology (Bethesda).

[20] Rabinowitz JD, Enerback S (2020). Lactate: the ugly duckling of energy metabolism. Nat Metab.

[21] Anders S, Huber W (2010). Differential expression analysis for sequence count data. Genome Biol.

[22] Wichmann G, Rosolowski M, Krohn K (2015). The role of HPV RNA transcription, immune response-related gene expression and disruptive TP53 mutations in diagnostic and prognostic profiling of head and neck cancer. Int J Cancer.

[23] Lohavanichbutr P, Mendez E, Holsinger FC (2013). A 13-gene signature prognostic of HPV-negative OSCC: discovery and external validation. Clin Cancer Res.

[24] Fountzilas E, Kotoula V, Angouridakis N (2013). Identification and validation of a multigene predictor of recurrence in primary laryngeal cancer. PLoS One.

[25] Yu G, Wang LG, Han Y (2012). clusterProfiler: an R package for comparing biological themes among gene clusters. OMICS.

[26] Gui J, Li H (2005). Penalized Cox regression analysis in the high-dimensional and low-sample size settings, with applications to microarray gene expression data. Bioinformatics.

[27] Hebert PD, Cywinska A, Ball SL (2003). Biological identifications through DNA barcodes. Proc Biol Sci.

[28] Pickett KL, Suresh K, Campbell KR (2021). Random survival forests for dynamic predictions of a time-to-event outcome using a longitudinal biomarker. BMC Med Res Methodol.

[29] Maeser D, Gruener RF, Huang RS (2021). oncoPredict: an R package for predicting in vivo or cancer patient drug response and biomarkers from cell line screening data. Brief Bioinform.

[30] Chen B, Khodadoust MS, Liu CL (2018). Profiling Tumor Infiltrating Immune Cells with CIBERSORT. Methods Mol Biol.

[31] Yoshihara K, Shahmoradgoli M, Martinez E (2013). Inferring tumour purity and stromal and immune cell admixture from expression data. Nat Commun.

[32] Sun J, Zhang Z, Bao S (2020). Identification of tumor immune infiltration-associated lncRNAs for improving prognosis and immunotherapy response of patients with non-small cell lung cancer. J Immunother Cancer.

[33] Li T, Fu J, Zeng Z (2020). TIMER2.0 for analysis of tumor-infiltrating immune cells. Nucleic Acids Res.

[34] Liu W, Wang Y, Bozi LHM (2023). Lactate regulates cell cycle by remodelling the anaphase promoting complex. Nature.

[35] Barrios-Bernal P, Hernandez-Pedro N, Orozco-Morales M (2022). Metformin Enhances TKI-Afatinib Cytotoxic Effect, Causing Downregulation of Glycolysis, Epithelial-Mesenchymal Transition, and EGFR-Signaling Pathway Activation in Lung Cancer Cells. Pharmaceuticals (Basel).

[36] Luo K, Liu A, Wu H (2022). CircKIF4A promotes glioma growth and temozolomide resistance by accelerating glycolysis. Cell Death Dis.

[37] Al-Ibraheem A, Mottaghy FM, Juweid ME (2023). PET/CT in Hodgkin Lymphoma: An Update. Semin Nucl Med.

[38] Brooks GA (2002). Lactate shuttles in nature. Biochem Soc Trans.

[39] Brooks GA (2018). The Science and Translation of Lactate Shuttle Theory. Cell Metab.

[40] Li X, Yang Y, Zhang B (2022). Lactate metabolism in human health and disease. Signal Transduct Target Ther.

[41] Zhang D, Tang Z, Huang H (2019). Metabolic regulation of gene expression by histone lactylation. Nature.

[42] Yu J, Chai P, Xie M (2021). Histone lactylation drives oncogenesis by facilitating m(6)A reader protein YTHDF2 expression in ocular melanoma. Genome Biol.

[43] Xiong J, He J, Zhu J (2022). Lactylation-driven METTL3-mediated RNA m(6)A modification promotes immunosuppression of tumor-infiltrating myeloid cells. Mol Cell.

[44] Colegio OR, Chu NQ, Szabo AL (2014). Functional polarization of tumour-associated macrophages by tumour-derived lactic acid. Nature.

[45] Bohn T, Rapp S, Luther N (2018). Tumor immunoevasion via acidosis-dependent induction of regulatory tumor-associated macrophages. Nat Immunol.

[46] Comito G, Iscaro A, Bacci M (2019). Lactate modulates CD4(+) T-cell polarization and induces an immunosuppressive environment, which sustains prostate carcinoma progression via TLR8/miR21 axis. Oncogene.

[47] Akaike T, Ida T, Wei FY (2017). Cysteinyl-tRNA synthetase governs cysteine polysulfidation and mitochondrial bioenergetics. Nat Commun.

[48] Coughlin CR 2nd, Scharer GH, Friederich MW (2015). Mutations in the mitochondrial cysteinyl-tRNA synthase gene, CARS2, lead to a severe epileptic encephalopathy and complex movement disorder. J Med Genet.

[49] Kropp PA, Wu J, Reidy M (2021). Allele-specific mitochondrial stress induced by Multiple Mitochondrial Dysfunctions Syndrome 1 pathogenic mutations modeled in Caenorhabditis elegans. PLoS Genet.

[50] Michl J, Wang Y, Monterisi S (2022). CRISPR-Cas9 screen identifies oxidative phosphorylation as essential for cancer cell survival at low extracellular pH. Cell Rep.

[51] Belenguer A, Duncan SH, Holtrop G (2007). Impact of pH on lactate formation and utilization by human fecal microbial communities. Appl Environ Microbiol.

[52] Bi M, Zheng L, Chen L (2021). ln RNA LINC01234 promotes triple-negative breast cancer progression through regulating the miR-429/SYNJ1 axis. Am J Transl Res.

