# Supplementary material for: Identification of a Lactate Accumulation Model to Explain the Heterogeneity in Prognosis, Immune Landscape, and Tumor Environment for HNSCC patients
Source: J Cancer. 2025 Feb 10;16(5):1668–83. doi: 10.7150/jca.99560 (PMC11843242; doi:10.7150/jca.99560)
Supplement: Supplementary file 1 — Supplementary figures and tables. [file jcav16p1668s1.pdf]

Supplementary Materials

Supplementary Materials

Figure S1

Consensus clustering of all HNSCC with k from 2 to 8.

Figure S2

The lactate-related somatic mutations in cluster_A and B. (A) The variants classification, types, SNV class and top mutated genes in HNSCC. (B) The waterfall plot showing the lactate-related genes related to cluster_A in HNSCC. (C) The waterfall plot showing the lactate-related genes related to cluster_B in HNSCC.

Figure S3

Construction the risk model based on LRGS. (A) The LASSO regression to select 10 LRGs to construct a risk model. (B) The ROC analysis evaluating the predictive ability of LRGS from RFS based on clusters when compared to other clinical characteristics. (C) The ROC analysis evaluating the predictive ability of LRGS from RFS based on survival status when compared to other clinical characteristics.

Figure S4

The drug sensitivity and the response to PD1/PD-L1 based on cluster_A and B. (A) The drug sensitivity in cluster_A or B using the OncoPredict algorithm. (B) The response to PD1/PD-L1 in cluster_A or B based on TCIA.

Figure S5

The distribution of immune cell types and immune-related scores from CIBERSORT and ESTIMATE in cluster_A or B.

Figure S6 Unraveling innate mechanism based on cluster_A or B. (A) The volcano plot showing the DEGs based on cluster_A or B. (B) The main pathways from the

GSEA enrichment analysis in comparison of cluster_A or B.

Figure S7 Validation the prognostic ability of hub genes in GEO datasets. (A) The Kaplan-Meier curves of OS for CARS2, NFU1, and SYNJ1 in GSE65858. (B) The Kaplan-Meier curves of OS for CARS2, NFU1, and SYNJ1 in GSE41613. (C) The Kaplan-Meier curves of PFS for CARS2, NFU1, and SYNJ1 in GSE27020. (D) The Kaplan-Meier curves of PFS for CARS2, NFU1, and SYNJ1 in GSE65858.

Figure S8 Validation the prognostic ability of hub genes in IMvigor 210. (A) The Kaplan-Meier curves of OS for CARS2, NFU1, and SYNJ1 in IMvigor 210. (B) The box plot displayed the expression of CARS2, NFU1, and SYNJ1 in diverse overall responses. (C) The box plot displayed the expression of CARS2, NFU1, and SYNJ1 in diverse immune phenotypes.

Figure S9 The relationship between hub genes and important clinical factors. (A) CARS2. (B) NFU1. (C) SYNJ1. (D) The innotation of clinical factors.

Table S1

The gene lists of nine classic lactate metabolism pathways in MSigDB and the two groups of lactate-related genes related to cluster_A and B.

.

Table S2

The coefficient of six crucial genes for calculating LRGS.

Table S3

The coefficient of nine tumor-infiltrating immune-related lncRNAs for calculating TIL_score.

Table S4

The gene lists of prognostic LRGs from Venn plot.

Table S5

The hub gene in HNSCC cells treated with lactate.

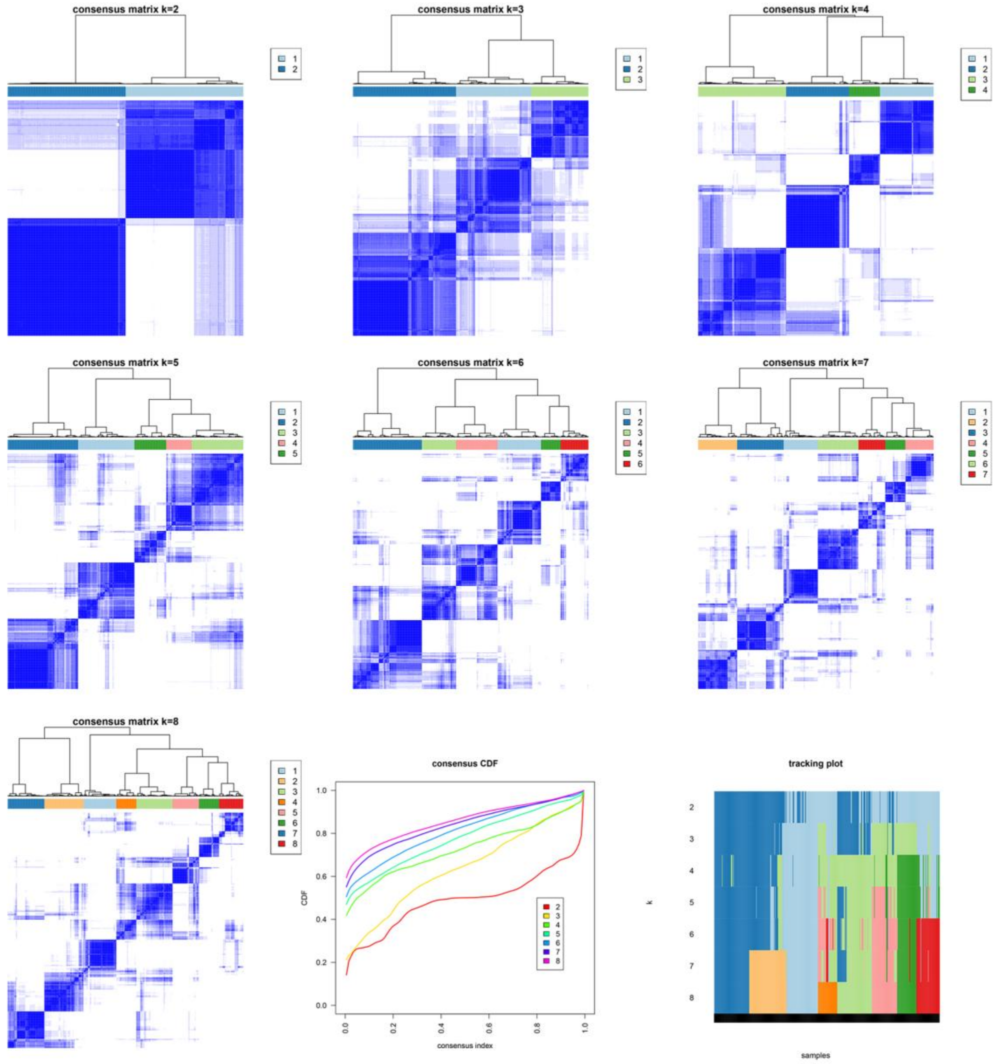

consensus matrix k=2
consensus matrix k=3
consensus matrix k=4
consensus matrix k=5
consensus matrix k=6
consensus matrix k=7
consensus matrix k=8
consensus CDF
CDF
consensus index
tracking plot
k
samples

Figure S1

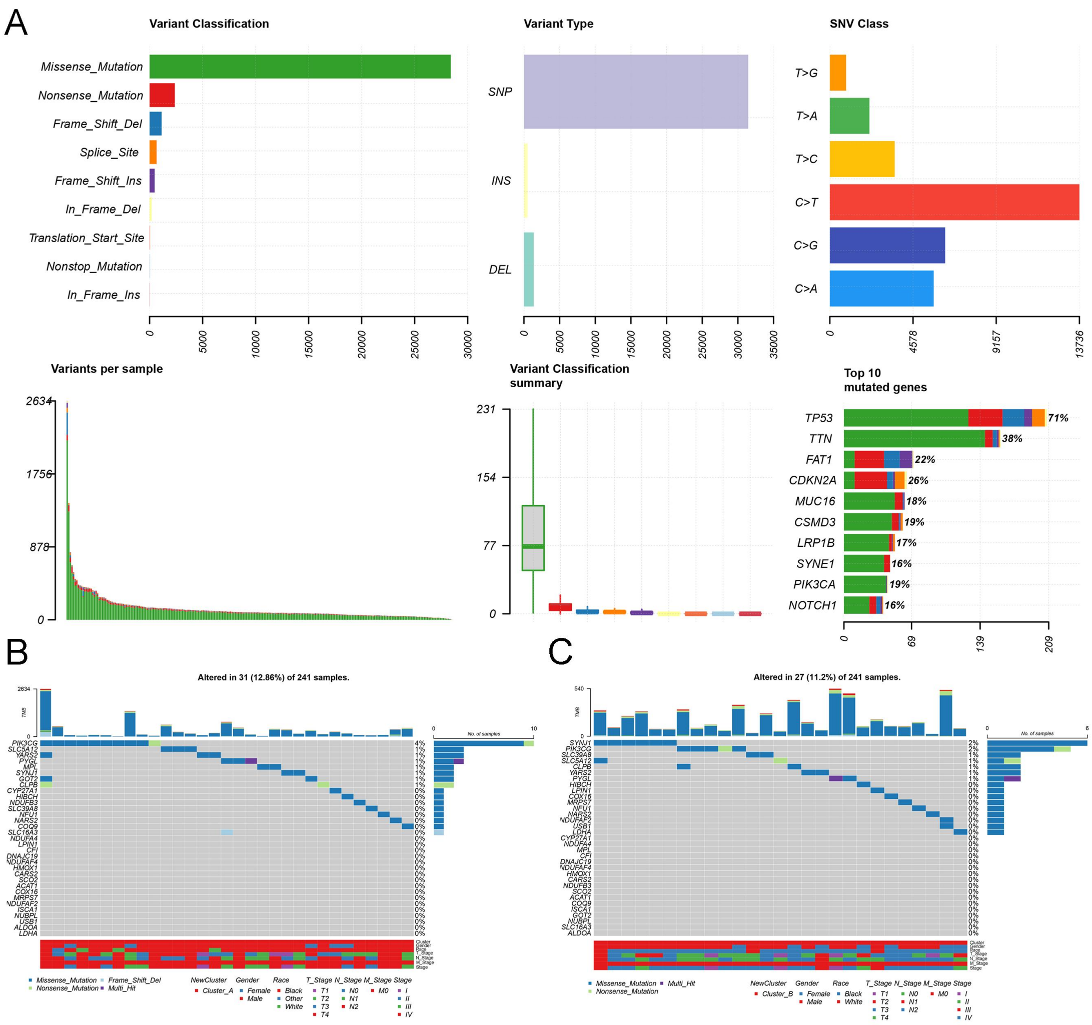
A
Variant Classification
Variant Type
SNV Class
Missense_Mutation
Nonsense_Mutation
Frame_Shift_Del
Splice_Site
Frame_Shift_Ins
In_Frame_Del
Translation_Start_Site
Nonstop_Mutation
In_Frame_Ins
SNP
INS
DEL
T>G
T>A
T>C
C>T
C>G
C>A
Variants per sample
Variant Classification summary
Top 10 mutated genes
TP53 71%
TTN 38%
FAT1 22%
CDKN2A 26%
MUC16 18%
CSMD3 19%
LRP1B 17%
SYNE1 16%
PIK3CA 19%
NOTCH1 16%
B
Altered in 31 (12.86%) of 241 samples.
C
Altered in 27 (11.2%) of 241 samples.

Figure S2

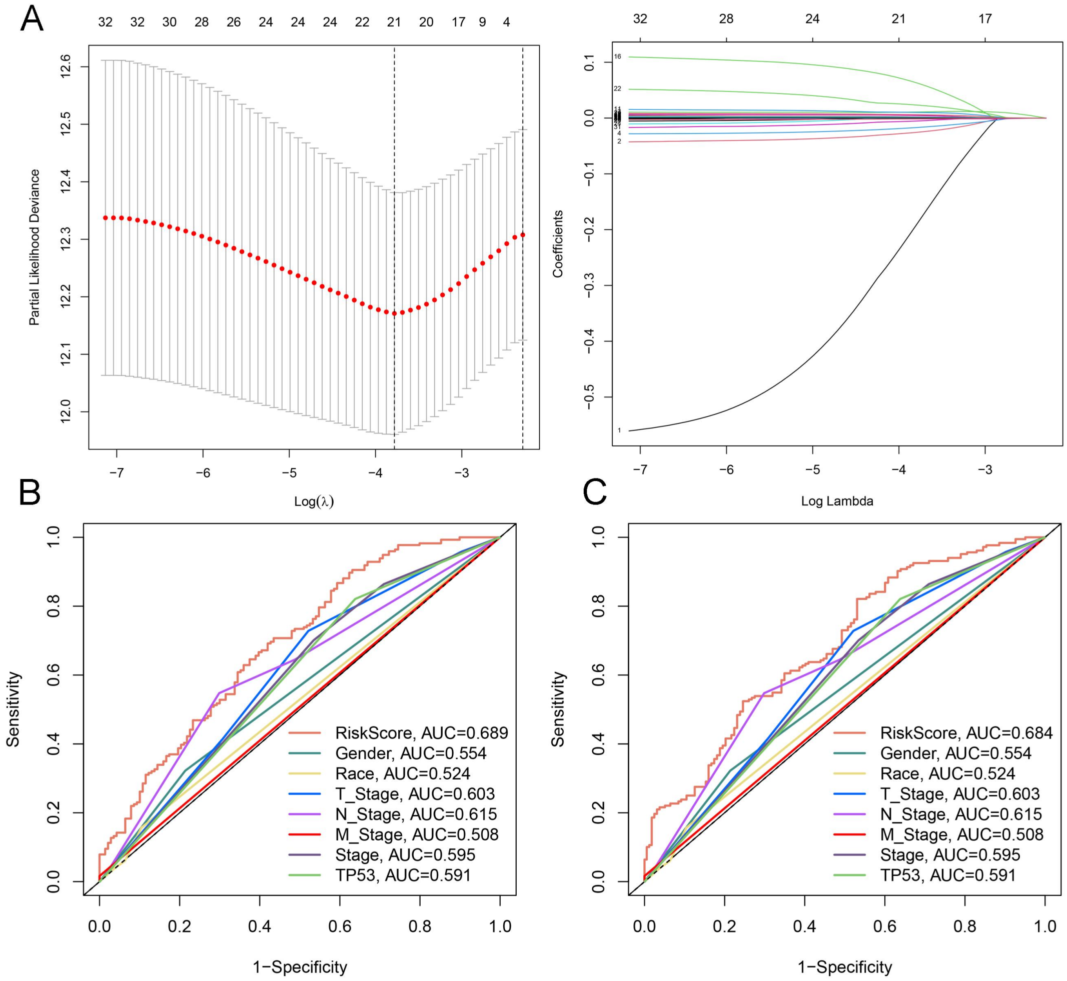
A
B
C
Partial Likelihood Deviance
Log(λ)
Coefficients
Log Lambda
Sensitivity
1−Specificity
RiskScore, AUC=0.689
Gender, AUC=0.554
Race, AUC=0.524
T_Stage, AUC=0.603
N_Stage, AUC=0.615
M_Stage, AUC=0.508
Stage, AUC=0.595
TP53, AUC=0.591
RiskScore, AUC=0.684
Gender, AUC=0.554
Race, AUC=0.524
T_Stage, AUC=0.603
N_Stage, AUC=0.615
M_Stage, AUC=0.508
Stage, AUC=0.595
TP53, AUC=0.591

Figure S3

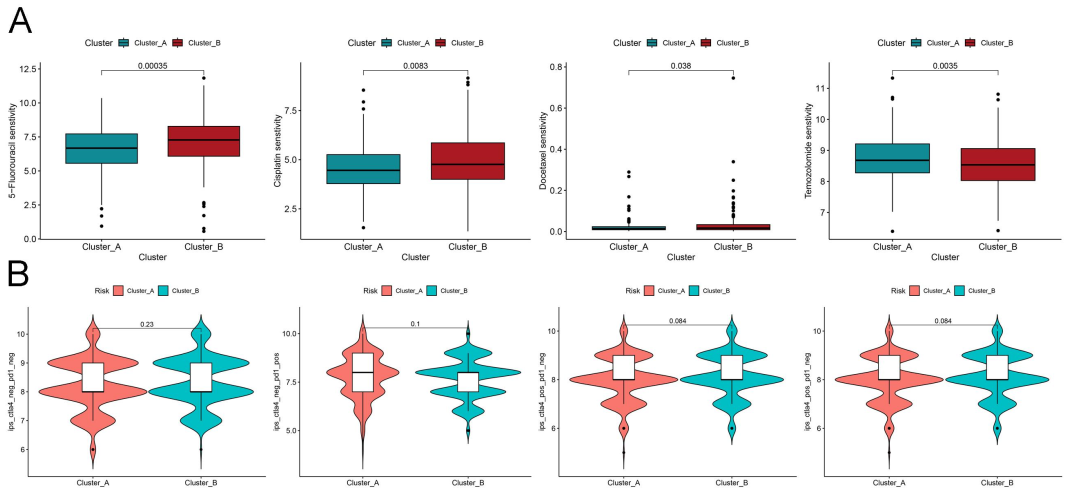
A
Cluster Cluster_A Cluster_B
0.00035
5−Fluorouracil sensitivity
Cluster_A Cluster_B
Cluster
0.0083
Cisplatin sensitivity
0.038
Docetaxel sensitivity
0.0035
Temozolomide sensitivity
B
Risk Cluster_A Cluster_B
0.23
ips_ctla4_neg_pd1_neg
0.1
ips_ctla4_neg_pd1_pos
0.084
ips_ctla4_pos_pd1_neg
ips_ctla4_pos_pd1_neg

Figure S4

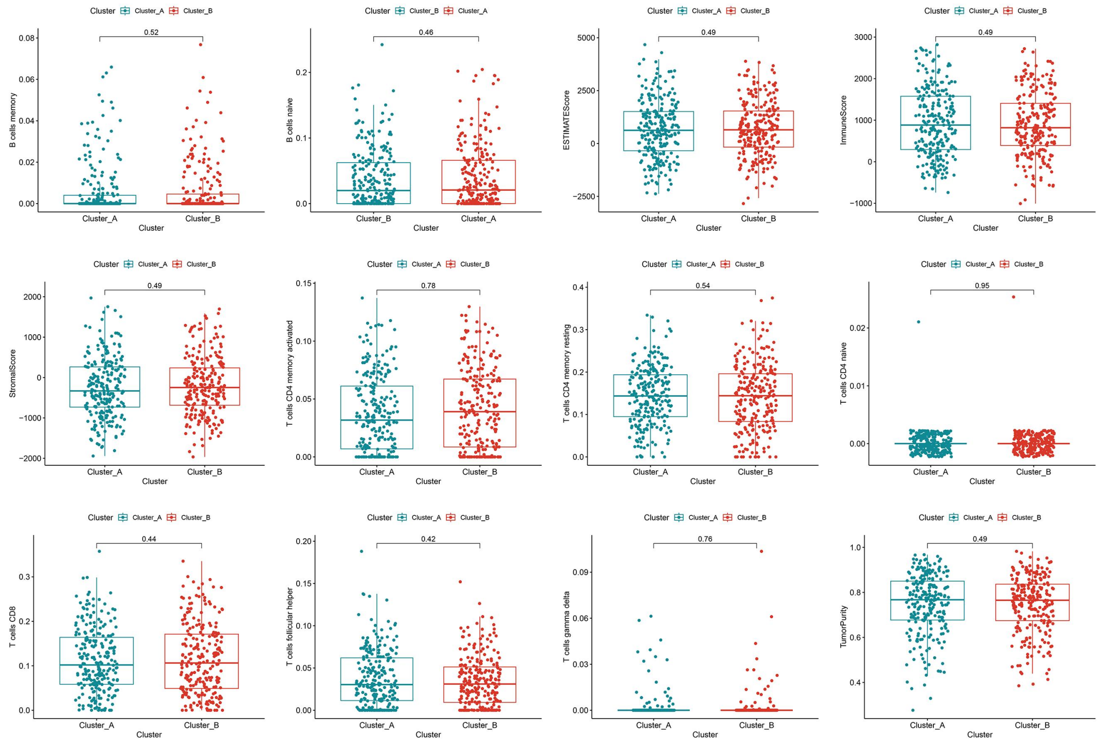
Cluster
Cluster_A
Cluster_B
B cells memory
B cells naive
ESTIMATEScore
ImmuneScore
StromalScore
T cells CD4 memory activated
T cells CD4 memory resting
T cells CD4 naive
T cells CD8
T cells follicular helper
T cells gamma delta
TumorPurity
0.52
0.46
0.49
0.49
0.49
0.78
0.54
0.95
0.44
0.42
0.76
0.49

Figure S5

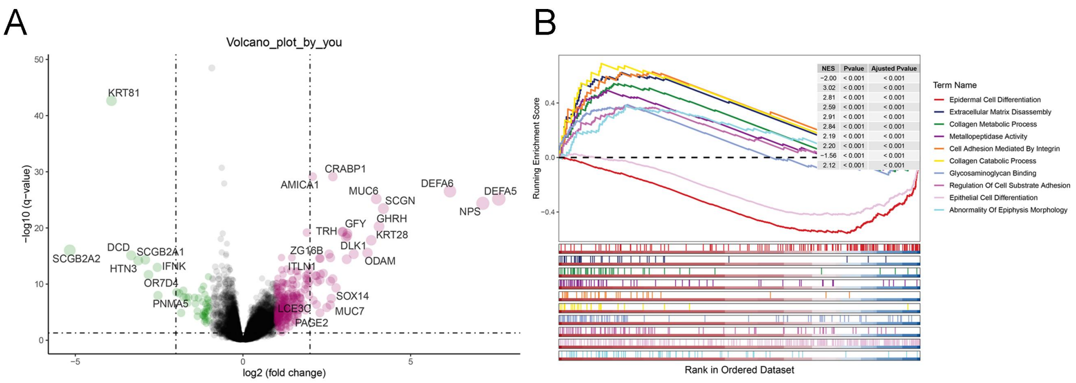
A
Volcano_plot_by_you
−log10 (q−value)
log2 (fold change)
KRT81
SCGB2A2
DCD
SCGB2A1
HTN3
IFNK
OR7D4
PNMA5
CRABP1
AMICA1
MUC6
SCGN
DEFA6
DEFA5
NPS
GHRH
GFY
TRH
KRT28
DLK1
ODAM
ZG16B
ITLN1
SOX14
MUC7
LCE3C
PAGE2
B
Running Enrichment Score
Rank in Ordered Dataset
NES
Pvalue
Ajusted Pvalue
−2.00 < 0.001 < 0.001
3.02 < 0.001 < 0.001
2.81 < 0.001 < 0.001
2.59 < 0.001 < 0.001
2.91 < 0.001 < 0.001
2.84 < 0.001 < 0.001
2.19 < 0.001 < 0.001
2.20 < 0.001 < 0.001
−1.56 < 0.001 < 0.001
2.12 < 0.001 < 0.001
Term Name
Epidermal Cell Differentiation
Extracellular Matrix Disassembly
Collagen Metabolic Process
Metallopeptidase Activity
Cell Adhesion Mediated By Integrin
Collagen Catabolic Process
Glycosaminoglycan Binding
Regulation Of Cell Substrate Adhesion
Epithelial Cell Differentiation
Abnormality Of Epiphysis Morphology

Figure S6

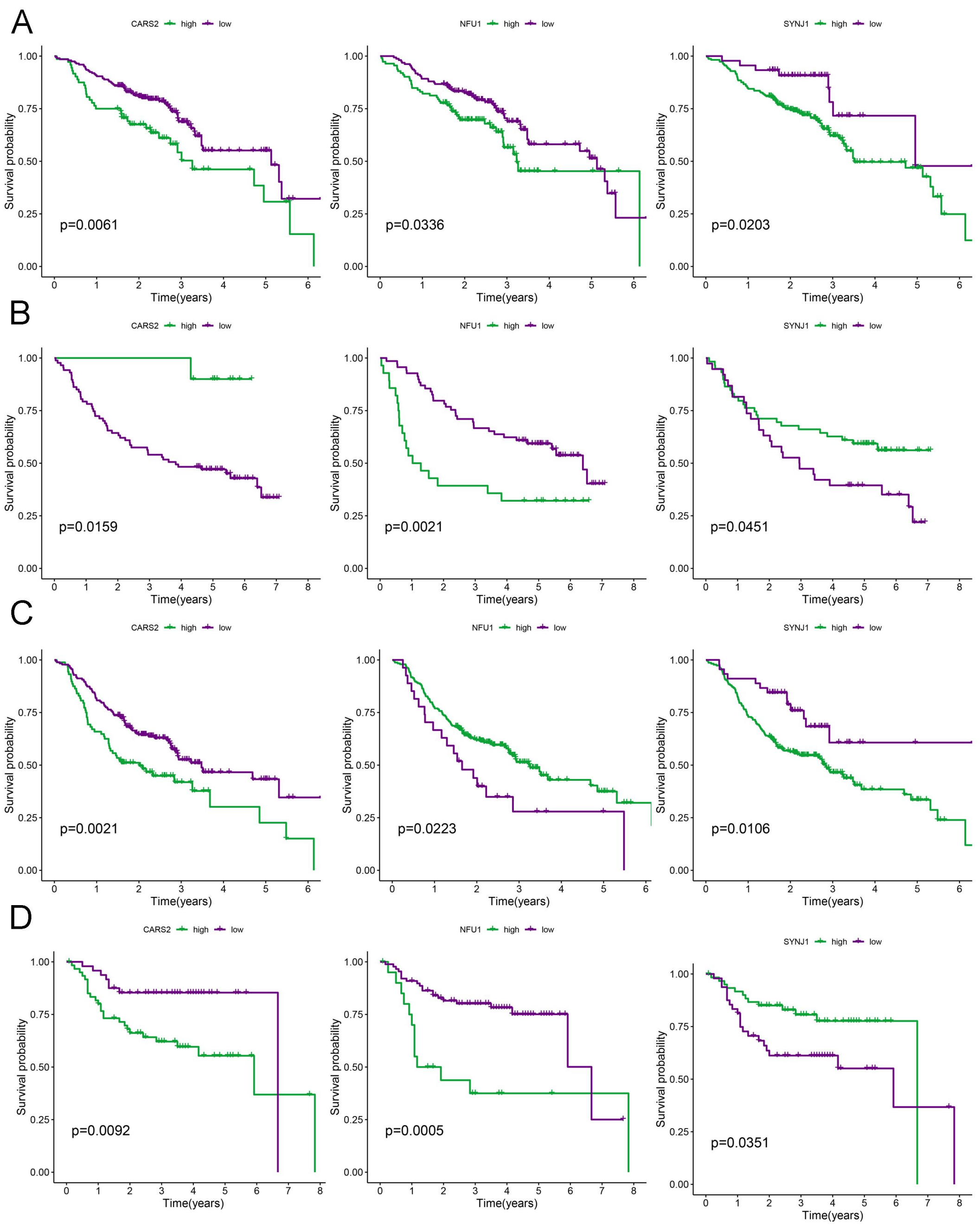
A
B
C
D
CARS2 high low
NFU1 high low
SYNJ1 high low
Survival probability
Time(years)
p=0.0061
p=0.0336
p=0.0203
p=0.0159
p=0.0021
p=0.0451
p=0.0021
p=0.0223
p=0.0106
p=0.0092
p=0.0005
p=0.0351

Figure S7

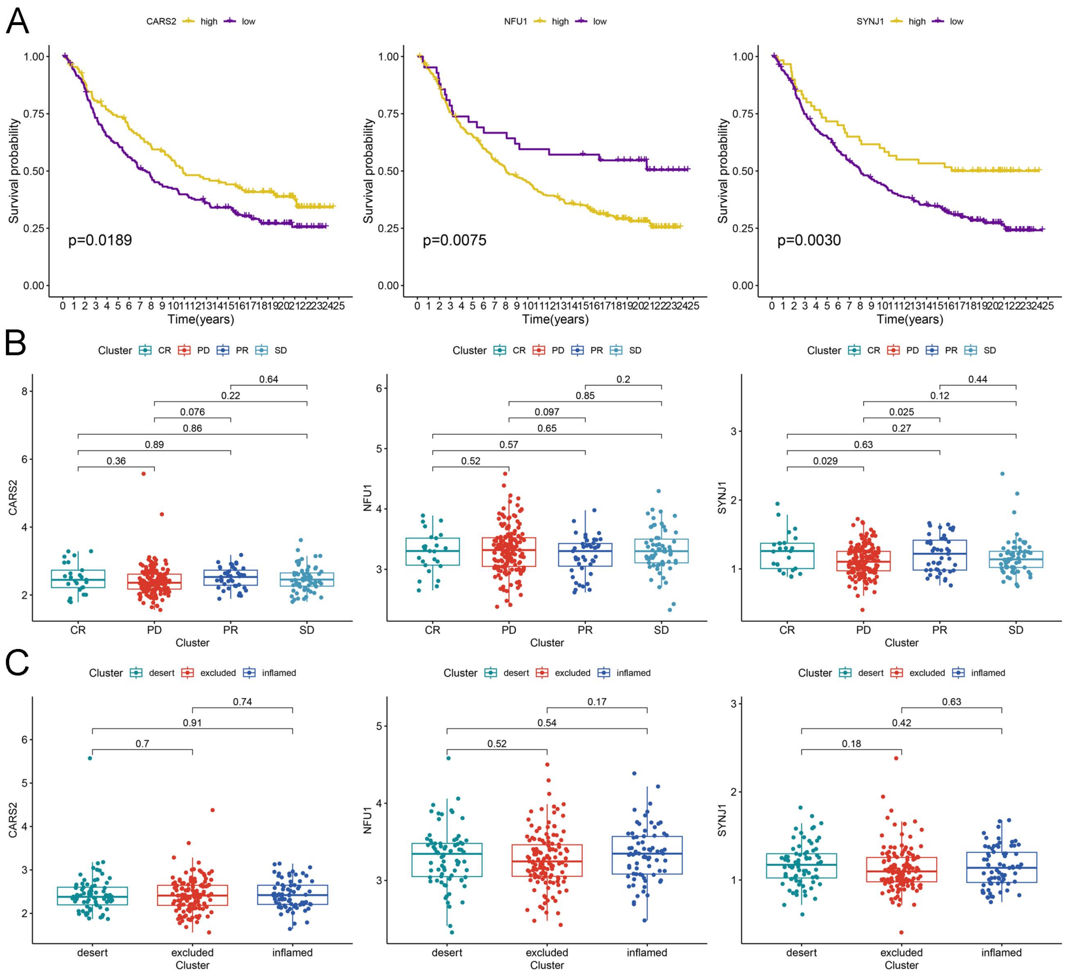

A
CARS2 high low
NFU1 high low
SYNJ1 high low
Survival probability
Time(years)
p=0.0189
p=0.0075
p=0.0030
B
Cluster CR PD PR SD
CARS2
NFU1
SYNJ1
Cluster
C
Cluster desert excluded inflamed
desert
excluded
inflamed

Figure S8

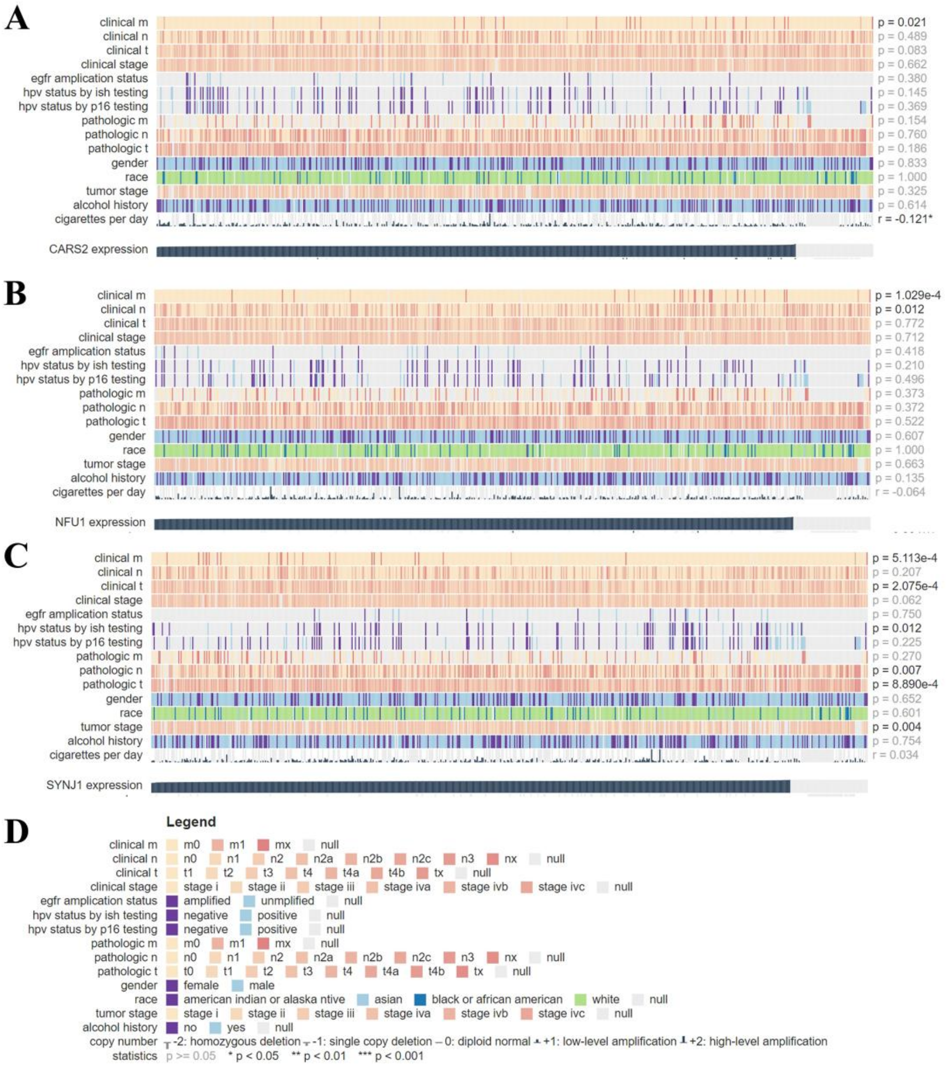
A
clinical m
clinical n
clinical t
clinical stage
egfr amplication status
hpv status by ish testing
hpv status by p16 testing
pathologic m
pathologic n
pathologic t
gender
race
tumor stage
alcohol history
cigarettes per day
p = 0.021
p = 0.489
p = 0.083
p = 0.662
p = 0.380
p = 0.145
p = 0.369
p = 0.154
p = 0.760
p = 0.186
p = 0.833
p = 1.000
p = 0.325
p = 0.614
r = -0.121*
CARS2 expression
B
p = 1.029e-4
p = 0.012
p = 0.772
p = 0.712
p = 0.418
p = 0.210
p = 0.496
p = 0.373
p = 0.372
p = 0.522
p = 0.607
p = 1.000
p = 0.663
p = 0.135
r = -0.064
NFU1 expression
C
p = 5.113e-4
p = 0.207
p = 2.075e-4
p = 0.062
p = 0.750
p = 0.012
p = 0.225
p = 0.270
p = 0.007
p = 8.890e-4
p = 0.652
p = 0.601
p = 0.004
p = 0.754
r = 0.034
SYNJ1 expression
D
Legend
clinical m: m0 m1 mx null
clinical n: n0 n1 n2 n2a n2b n2c n3 nx null
clinical t: t1 t2 t3 t4 t4a t4b tx null
clinical stage: stage i stage ii stage iii stage iva stage ivb stage ivc null
egfr amplication status: amplified unmplified null
hpv status by ish testing: negative positive null
hpv status by p16 testing: negative positive null
pathologic m: m0 m1 mx null
pathologic n: n0 n1 n2 n2a n2b n2c n3 nx null
pathologic t: t0 t1 t2 t3 t4 t4a t4b tx null
gender: female male
race: american indian or alaska ntive asian black or african american white null
tumor stage: stage i stage ii stage iii stage iva stage ivb stage ivc null
alcohol history: no yes null
copy number: -2: homozygous deletion -1: single copy deletion 0: diploid normal +1: low-level amplification +2: high-level amplification
statistics: p >= 0.05 * p < 0.05 ** p < 0.01 *** p < 0.001

Figure S9

**MSigDB from lactate accumulation**							**Poor OS**	**Good OS**	**Cluster_A**	**Cluster_B**
SLC5A12	SUCLG1	INPP5K	LRPPRC	DARS2	COQ2	MRPS14	SLC5A12	CYP27A1	CYP27A1	SLC39A8
SLC5A8	LYRM7	PIGA	COX20	FOXRED1	GOT2	MRPL44	SLC16A3	SCO2	NDUFA4	ACAT1
SLC16A8	MTRFR	PIK3CG	MTFMT	TMEM126B	SLC25A42	SOD1	SLC39A8	FLI1	HIBCH	SLC5A12
SLC16A1	SCO2	PLEC	NDUFA11	NDUFA12	MRPS28	STAT2	CFI	LPIN1	LPIN1	COX16
SLC16A3	HELLPAR	TET2	NAXE	COQ8A	TIMM22	C1QBP	IRAK1	MPL	MPL	MRPS7
SLC16A7	SLC25A13	NSUN2	COX4I1	RARS2	GYS2	TK2	USB1	PIK3CG	PIK3CG	NFU1
LDHAL6A	TCIRG1	POMGNT1	COX6B1	SLC25A19	HMGCL	TUFM	COX20	PLA2G6	SYNJ1	NARS2
LDHD	RXYLT1	PNPLA2	COX8A	SLC13A3	ACAT1	UQCRB	COX15	SYNJ1	CFI	COQ9
LDHA	PITRM1	ZNFX1	COX10	SLC2A1	LIPT2	UQCRC2	NFU1		DNAJC19	NDUFAF2
LDHB	POMT1	RB1	COX15	NDUFAF5	ACAT2	UQCRH	NDUFA4		NDUFAF4	ISCA1
LDHAL6B	KLF1	RHAG	TXN2	NARS2	LETM1	CA5A	DARS2		HMOX1	GOT2
DNM1L	B4GAT1	RHCE	TPK1	NUBPL	CHCHD10	UQCC3	RARS2		CARS2	NUBPL
FARS2	CHEK2	RHD	NFU1	PDHX	MIPEP	CARS2	SLC13A3		NDUFB3	CLPB
NDUFAF6	LYST	RPS14	NDUFAF8	SLC19A3	ALDH6A1	PPCS	NARS2		SCO2	YARS2
CYP27A1	COL4A1	ABCG8	NDUFAF4	CLPB	MT-TI	TTC26	NUBPL			USB1
ECHS1	CPT2	SIL1	SLC25A4	UQCC2	MT-TL2	TARS2	CLPB			PYGL
NDUFAF3	CDAN1	SLC4A1	NDUFS7	COA8	MT-TP	PUS1	AIFM1			SLC16A3
HTRA2	B3GALNT2	SLC19A1	MECP2	GFM2	NDUFC2	ISCA1	DNAJC19			ALDOA
HSD17B10	DAG1	SPP1	MPV17	COX14	OGDH	GTPBP3	GOT2			LDHA
KARS1	FKTN	AKR1D1	MT-ATP6	ALDH4A1	PNPLA8	SERAC1	ACAT1			
MT-CO1	ALDOA	STAT4	MT-ND2	PNPT1	PC	YARS1	YARS2			
MT-CO2	FLI1	TP53	MT-ND3	AIFM1	MRPS16	SLC25A12	COQ9			
MT-CO3	LPIN1	CRPPA	MT-TK	ATPAF2	YARS2	SYNJ1	PYGL			
MT-ND1	VPS13A	XK	MT-TN	NDUFAF2	MRPS7	HS6ST2	CARS2			

MT-ND4	GAA	FKRP	MT-TV	TIMM50	MRPS2	NFS1	TARS2
MT-ND5	GATA1	USB1	NDUFA1	LONP1	COQ4	COX5A	ISCA1
MT-ND6	GATA2	CALR	NDUFA2	WARS2	MPC1	PMPCB	
MT-TF	CBLIF	PLA2G6	NDUFA4	LIAS	UPB1		
MT-TH	ACAD9	POMK	NDUFA6	ADAMTS13	SLC25A3		
MT-TL1	POMT2	POMGNT2	NDUFA8	PLPBP	PHKG2		
MT-TQ	HBB	CAV3	NDUFA10	POLG2	POLG		
MT-TS2	CFH	MLIP	NDUFB3	FDX2	PDP1		
MT-TW	HLA-DRB1	SLC7A7	NDUFB9	SLC25A26	TMEM70		
NDUFB7	HMGCS2	LARGE1	NDUFS1	SFXN4	PNPO		
NDUFB8	HMOX1	KIF23	NDUFS3	EARS2	ATAD3A		
NDUFS2	KY	PIEZO1	NDUFV1	MICOS13	TRMU		
MECR	ACADM	TSFM	NDUFS6	TANGO2	NAXD		
COX16	CFI	MRPL3	NDUFS8	DNAJC19	AGK		
POLRMT	IRAK1	CYC1	NDUFV2	COX6A2	NGLY1		
LYRM4	JAK2	PDSS1	RRM2B	SLC25A10	TWNK		
VARS2	KCNN4	MDH2	NDUFA13	DGUOK	MRPS22		
SQOR	LIPA	NDUFA9	NDUFAF1	DLD	COQ9		
SDHB	CD46	NDUFB10	TRAPPC12	DTYMK	PDSS2		
SLC39A8	MPL	NDUFS4	TACO1	FASTKD2	AARS2		
MRPS34	MTHFD1	BCS1L	TIMMDC1	ISCU	TRMT5		
SURF1	MVK	SDHA	PDHA1	RNASEH1	PYGL		
COG8	MYC	GFM1	LIPT1	MTO1	RARS1		
HPDL	OCRL	PRORP	NDUFB11	FBXL4	MRPL12		
SUCLG1	INPP5K	PET100	TRMT10C	HIBCH	SCO1		
LYRM7	PIGA	PET117	RMND1	UQCRQ	NSUN3		

Table S1

Gene	Coef
MPL	-0.187781556
LPIN1	-0.025384765
NFU1	0.010898831
HIBCH	-0.016822569
NDUFB3	0.000769955
CYP27A1	-0.001611948
SLC39A8	0.001117786
CFI	0.004975383
NDUFAF4	0.010080619
SLC5A12	0.062473431
ACAT1	0.004607103
CARS2	0.006013107
NUBPL	0.023182463
PYGL	0.000547012
ALDOA	0.000339325
USB1	0.002425176
GOT2	0.000639595
SLC16A3	0.001605662
SYNJ1	-0.005200659
HMOX1	0.000225916
SCO2	-0.001516521

Table S2

id	coef	HR	HR.95L	HR.95H	pvalue
ITPKB-IT1	-0.161002722	0.851289754	0.765301916	0.946939019	0.003041647
SNHG15	-0.049266525	0.951927383	0.910311677	0.995445588	0.03076415
PCOLCE-AS1	-0.060880193	0.940935964	0.879822909	1.006293971	0.075593855
SNHG6	0.039824613	1.040628246	0.996279896	1.086950716	0.073095301
WAC-AS1	-0.060438722	0.941351451	0.89747141	0.987376918	0.013081078
TMEM254-AS1	0.038920191	1.039687504	0.991335128	1.090398267	0.109200132
NUTM2A-AS1	0.047952284	1.049120594	0.998906145	1.101859295	0.055336035
VPS9D1-AS1	0.044099557	1.045086395	1.000583651	1.091568478	0.04700624
ITGB2-AS1	-0.049858787	0.95136376	0.906287938	0.998681507	0.044089568

Table S3

TCGA	GSE65858	GSE41613	GSE27070 PFS	GSE65858 PFS
MPL	GOT2	NUBPL	NFU1	SLC16A3
LPIN1	SLC16A3	USB1	LPIN1	HIBCH
NFU1	ACAT1	HIBCH	SCO2	HMOX1
HIBCH	CARS2	NFU1	CARS2	CARS2
NDUFB3	HMOX1	ACAT1	NDUFAF4	GOT2
CYP27A1	ALDOA	NDUFB3	NUBPL	CFI
SLC39A8	MPL	SCO2	SYNJ1	LPIN1
CFI	HIBCH	MPL	GOT2	ACAT1
NDUFAF4	SYNJ1	CARS2	MPL	SYNJ1
SLC5A12	NFU1	SLC39A8		NFU1
ACAT1	NDUFB3	PYGL		SLC39A8
CARS2		ALDOA		ALDOA
NUBPL		SLC5A12		
PYGL		SYNJ1		
ALDOA		HMOX1		
USB1				
GOT2				
SLC16A3				
SYNJ1				
HMOX1				
SCO2				

Table S4

gene_name	logFC	AveExpr	t	P.Value	adj.P.Val	B
CARS2	-2.492630409	9.143093418	-17.58409655	1.80E-05	0.000896265	3.55075874
SYNJ1	0.33924216	1.0002662	5.393943076	0.003556666	0.019718563	-2.708848056
NFU1	1.499525671	17.18755313	2.422781145	0.063069709	0.169208894	-6.014724051

Table S5
